# ASYNAPSIS 1 ensures crossover fidelity in polyploid wheat by promoting homologous recombination and suppressing non-homologous recombination

**DOI:** 10.3389/fpls.2023.1188347

**Published:** 2023-05-22

**Authors:** Chiara Di Dio, Heïdi Serra, Pierre Sourdille, James D. Higgins

**Affiliations:** ^1^ Department of Genetics and Genome Biology, Adrian Building, University of Leicester, Leicester, United Kingdom; ^2^ Genetics, Diversity and Ecophysiology of Cereals, Unité Mixte de Recherche (UMR) 1095, The Institut National de la Recherche Agronomique (INRAE), Université Clermont Auvergne, Clermont-Ferrand, France

**Keywords:** chromosomes, chiasma, homoeologous, meiosis, synapsis, introgression

## Abstract

During meiosis, the chromosome axes and synaptonemal complex mediate chromosome pairing and homologous recombination to maintain genomic stability and accurate chromosome segregation. In plants, ASYNAPSIS 1 (ASY1) is a key component of the chromosome axis that promotes inter-homolog recombination, synapsis and crossover formation. Here, the function of ASY1 has been cytologically characterized in a series of hypomorphic wheat mutants. In tetraploid wheat, *asy1* hypomorphic mutants experience a reduction in chiasmata (crossovers) in a dosage-specific manner, resulting in failure to maintain crossover (CO) assurance. In mutants with only one functional copy of ASY1, distal chiasmata are maintained at the expense of proximal and interstitial chiasmata, indicating that ASY1 is required to promote chiasma formation away from the chromosome ends. Meiotic prophase I progression is delayed in asy1 hypomorphic mutants and is arrested in *asy1* null mutants. In both tetraploid and hexaploid wheat, single asy1 mutants exhibit a high degree of ectopic recombination between multiple chromosomes at metaphase I. To explore the nature of the ectopic recombination, *Triticum turgidum* asy1b-2 was crossed with wheat-wild relative *Aegilops variabilis*. Homoeologous chiasmata increased 3.75-fold in *Ttasy1b-2/Ae. variabilis* compared to wild type/*Ae. variabilis*, indicating that ASY1 suppresses chiasma formation between divergent, but related chromosomes. These data suggest that ASY1 promotes recombination along the chromosome arms of homologous chromosomes whilst suppressing recombination between non-homologous chromosomes. Therefore, *asy1* mutants could be utilized to increase recombination between wheat wild relatives and elite varieties for expediting introgression of important agronomic traits.

## Introduction

The majority of sexually reproducing eukaryotes undergo meiosis, a specialized cell division required to produce haploid gametes from diploid progenitor cells. Meiosis is characterized by the homologous recombination of genetic material between chromosomes that is necessary to ensure accurate chromosome segregation as well as create new combinations of alleles. In wheat, meiotic recombination is initiated by ~2,000 programmed DNA double-strand breaks (DSBs) (Gardiner et al., 2019), catalyzed by SPO11-1/SPO11-2 (Benyahya et al., 2020; Da Ines et al., 2020; Hyde et al., 2022). DSBs are repaired as crossovers (COs) when a reciprocal exchange of DNA takes place between homologous chromosomes (that are cytologically detected as chiasmata), or non-crossovers (NCOs) when DSBs are repaired by non-reciprocal exchange of DNA, *via* either the sister chromatid or homologous chromosome as a template. In plants, ~85% of COs form *via* the class I pathway that ensures every chromosome pair receives at least one “obligate CO” so that homologous chromosomes are tethered together at metaphase I and accurately segregate during meiosis II (Higgins et al., 2004; Higgins et al., 2008b; Osman et al., 2011). Class I COs are sensitive to interference and therefore more likely to be spaced apart than by random chance (Jones and Franklin, 2006). The class II pathway accounts for ~15% of COs and is insensitive to interference (Berchowitz et al., 2007; Higgins et al., 2008a; Lambing et al., 2017; Wang and Copenhaver, 2018; Desjardins S. D. et al., 2020). In wheat, the FANCM helicase promotes class I COs as well as suppressing class II CO formation, suggesting that the two CO pathways are intimately linked (Desjardins et al., 2022).

In plants, inter-homolog recombination and the obligate CO are promoted by the synaptonemal complex (SC), which also imposes CO interference (Higgins et al., 2005; Sanchez-Moran et al., 2007; Sanchez-Moran et al., 2008; Ferdous et al., 2012; Chambon et al., 2018; Capilla-Perez et al., 2021; France et al., 2021). The SC is an evolutionary conserved tripartite proteinaceous structure that assembles and disassembles during meiotic prophase I (Page and Hawley, 2004; Hughes and Hawley, 2020). The SC is composed of two chromosome axes that mature into lateral elements upon installation of the transverse filament proteins (Page and Hawley, 2004; Gao and Colaiacovo, 2018). The core components of the chromosome axes are ASYNAPSIS 1 (ASY1)/PAIR2, ASY3/PAIR3, and ASY4 (Armstrong et al., 2002; Nonomura et al., 2004; Yuan et al., 2009; Ferdous et al., 2012; Chambon et al., 2018) as well as the transverse filament proteins ZYP1/ZEP1 (Higgins et al., 2005; Wang et al., 2010; Barakate et al., 2014). ASY1 possesses a conserved HORMA domain that is predicted to bind to chromatin along with its interacting partners p31COMET, ASY3, and ASY4 (Caryl et al., 2000; Armstrong et al., 2002; Sanchez-Moran et al., 2007; Ferdous et al., 2012; Chambon et al., 2018; Balboni et al., 2020). ASY1 also acts as a gene dosage-dependent antagonist of telomere-led recombination in *Arabidopsis*, thereby promoting interfering COs (Lambing et al., 2020), although ASY1 immunoprecipitation experiments in wheat suggest that the protein is more abundant toward the chromosome ends (Tock et al., 2021).

Wheat is an allopolyploid crop in which COs predominantly form toward the chromosome ends (Saintenac et al., 2009; Osman et al., 2021; Higgins et al., 2022). It has evolved a meiotic program in which homoeologous chromosomes rarely recombine due to the *Pairing homoeologous* (*Ph*) *1* and *2* loci (Riley and Chapman, 1958; Mello-Sampayo, 1971). *TaZIP4-B2* gene in the *Ph1* locus is required for both promotion of homologous COs and restriction of homoeologous COs in wheat/*Aegilops variabilis* hybrids (Rey et al., 2017; Rey et al., 2018), while *TaMSH7-3D* in the *Ph2* locus is necessary for recombination partner selection (homologous *vs.* homoeologous) by likely increasing the instability of homoeologous recombination in wheat/*Ae. variabilis* hybrids (Serra et al., 2021). In addition, reduced expression of *ASY1* by RNAi in hexaploid wheat generated high levels of multiple chromosome configurations at metaphase I, implying loss of CO control and elevated homoeologous recombination (Boden et al., 2009).

Here, cytological analysis of hypomorphic wheat *asy1* TILLING (Targeting Induced Local Lesions In Genomes) mutants has revealed a delay in meiotic progression, loss of the obligate chiasma, and ectopic recombination between multiple chromosomes. Tetraploid wheat *asy1* mutants crossed with wheat-wild relative *Ae. variabilis* exhibit an increase in chiasma formation, indicating that ASY1 is dosage-sensitive for promoting accurate homologous recombination while suppressing non-homologous recombination during meiosis.

## Experimental procedures

### Plant material and greenhouse conditions

*Triticum turgidum* ‘Kronos’ and *Triticum aestivum* ‘Cadenza’ were used as wild-type controls for experiments involving TILLING mutant lines received from www.SeedStor.ac.uk. The Ensembl Plants database (http://plants.ensembl.org) was used to identify ASY1 genes: *TtASY1-5A*, TRITD5Av1G167820; *TtASY1-5B*, TRITD5Bv1G159710; *TaASY1-5A*, TraesCS5A02G286500; *TaASY1-5B*, TraesCS5B02G285800; and *TaASY1-5D*, TraesCS5D02G294100. TILLING mutants were screened by BLAST search on the Wheat TILLING database (http://www.wheat-tilling.com/): *Ttasy1a*, K0706; *Ttasy1b-1*, K0157; *Ttasy1b-2*, K2071 (Krasileva et al., 2017); and *Taasy1b*, C0971 (Appels et al., 2018). To create hypomorphic mutants, homozygous lines were crossed (K0706 *Ttasy1a* × K0157 *Ttasy1b-1* and K0706 *Ttasy1a* × K2071 *Ttasy1b-2*), while heterozygous individuals from the F1’s (AaBb) were self-pollinated to create F2’s. Wild-type Kronos and the *Ttasy1b-2* mutant line (K2071) were crossed with *Ae. variabilis* (accession no. 26248, https://www6.clermont.inrae.fr/umr1095_eng/Organisation/Experimental-Infrastructure/Biological-Resources-Centre; UUSS, 2n = 4x = 28) to produce Kronos/*Ae. variabilis* haploid hybrids (ABUS, n = 28). Briefly, Kronos inflorescences were emasculated and pollinated with fresh *Ae. variabilis* pollen. Inflorescences were then bagged to avoid cross-pollination, and seeds were collected when mature. Plants were grown in soil-based compost (Levington Advance Pot and Bedding M1 Compost) under greenhouse conditions with a photoperiod of 16-h days light cycle at a constant temperature of 22°C (day)/16°C (night) and relative humidity ~60%.

### Validating SNP mutations

To validate that the point mutations induced by ethyl methanesulfonate treatment would be transcribed into mRNA, total RNA was extracted from tetraploid wheat *T. turgidum* ‘Kronos’ and hexaploid *T. aestivum* ‘Cadenza’ inflorescences using the ISOLATE II RNA Mini Kit (https://www.bioline.com/). cDNA was synthesized using the Tetro cDNA Synthesis Kit (https://www.bioline.com/), followed by PCR with Q5^®^ High-Fidelity DNA Proofreading Polymerase (https://www.neb.uk.com/) with primers TaASY1cDNAF and TaASY1cDNAR (**Supplementary Table 1**). PCR amplicons were ligated into pDrive (https://www.qiagen.com/) and Sanger sequenced (https://eurofinsgenomics.eu/). Following validation, single-nucleotide polymorphism (SNP)-specific primers were designed to amplify individual TILLING lines for genotyping optimized by gradient PCR.

### Cytological procedures

Anther sizes were measured with a Nikon SMZ 745 dissecting microscope and 10 mm/0.1 mm graticule. Chromosome spreads were stained with DAPI and examined by light microscopy as previously described (Higgins, 2013; Desjardins S. et al., 2020). Nikon Ni-E and Eclipse Ci fluorescence microscopes equipped with NIS elements software were used to image chromosomes. The following primary antibodies were used for immunolocalization: anti-*Ta*ASY1 guinea pig, 1:500 (Desjardins S. D. et al., 2020); and anti-*At*ZYP1 rabbit 1:500 (Osman et al., 2018). Secondary antibodies: goat anti-guinea pig Alexa Fluor 488 (https://www.abcam.com/) and goat anti-rabbit DyLight 594 (https://www.2bscientific.com/) were used at 1:200. Chiasma counts were performed using NIS software, and significance (*p* adj < 0.01) was established using pairwise Wilcoxon rank sum tests adjusted with Bonferroni correction method (RStudio v1.2.5033). The karyology of *Kronos*/*Ae. variabilis* hybrids was checked by aceto-carmine chromosome spreads as previously described (Serra et al., 2021).

### Statistical analysis

A chi-square test for analysis of meiotic progression in *asy1* hypomorphic mutants was performed to test the association between meiotic prophase I stages and anther lengths, and a significant *p*-value was set less than 0.05. A statistical analysis of seed counts per plant comparing the primary inflorescence was performed on Minitab 20 with a *t*-test two-sample distribution.

## Results

### Identification of wheat ASY1

**Figure 1:**
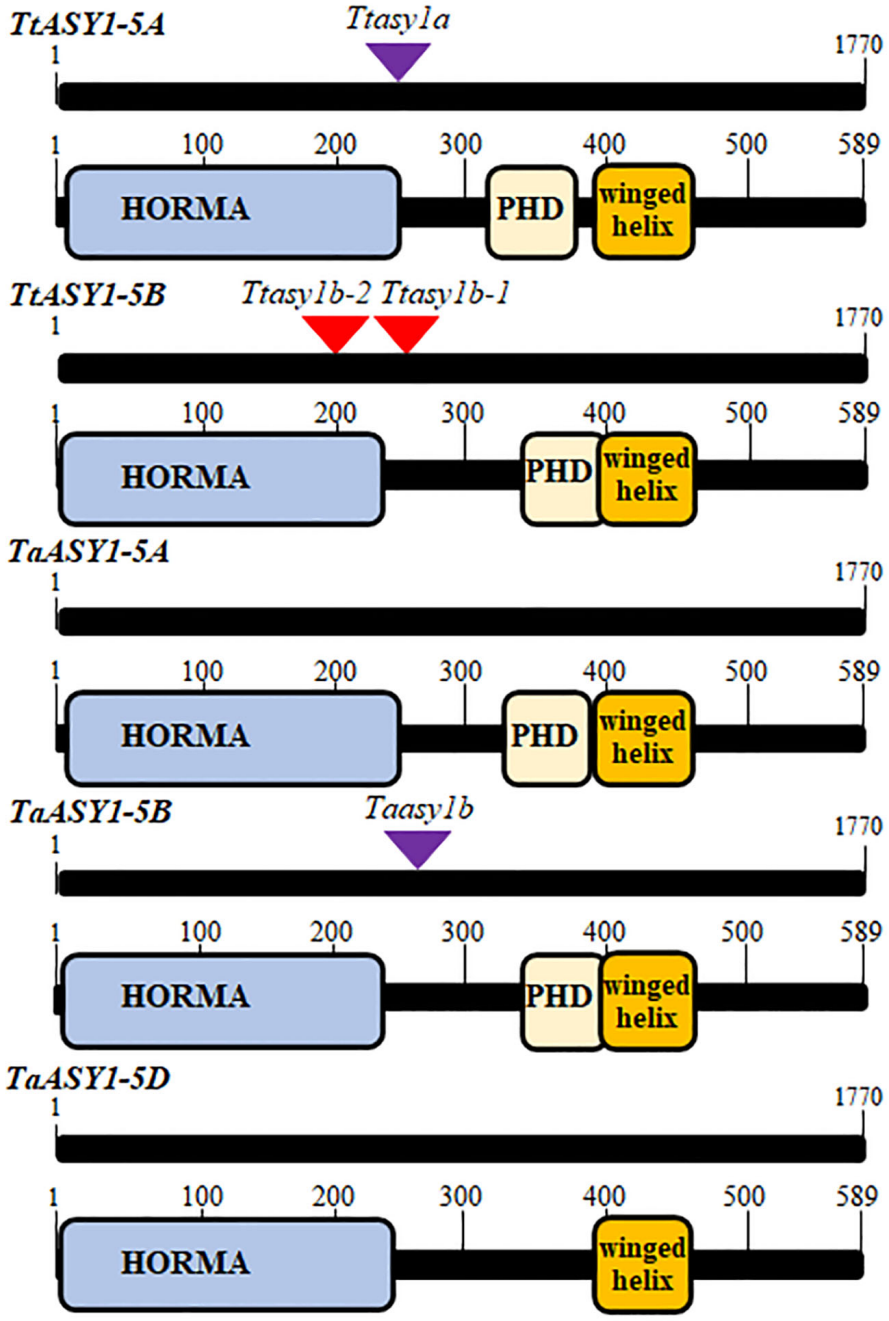
Schematic representation of wheat *ASY1* coding regions and altered proteins from TILLING mutations. The coding regions of *ASY1* and the Phyre2 predicted protein domains are shown relative to the TILLING mutations. The purple triangle represents a mutated splice donor site that retains an intron, whereas the red triangles represent a stop codon.

ASY1 is a component of the meiotic chromosome axis that is highly expressed in anthers during prophase I of meiosis (Boden et al., 2009; Alabdullah et al., 2019; Tock et al., 2021; Jiang et al., 2023). The wheat ASY1 coding sequences were cloned and sequenced from tetraploid ‘Kronos’ and hexaploid ‘Cadenza’ cDNA (**Supplementary Figure 1**). A wheat consensus ASY1 protein sequence derived from the clones shares a high level of sequence similarity to PAIR2 in *Oryza sativa* (80%), ASY1 in *Arabidopsis thaliana* (54%), and ASY1 in *Brassica oleracea* (51%). The polyploid wheat ASY1 homoeologous sequences share >96% nucleotide identity and >94% amino acid identity (**Supplementary Table 2**). ASY1 is located on the long arm of chromosome 5 in tetraploid wheat and hexaploid wheat. A Phyre2 structural analysis (Kelley et al., 2015) predicts that the wheat ASY1 proteins contain a conserved N-terminal HORMA domain (100% prediction at residues 6–236 for 5A and 3–231 for 5B and 5D) and a winged helix DNA binding domain (96% prediction at residues 339–457) (**Figure 1** and **Supplementary Table 3**). ASY1-5A and ASY1-5B are predicted to contain a Set3 PhD finger H3K4me3 domain (85%–91% prediction for ASY1-5A and 21%–42% ASY1-5B at residues 317–400) but not detected in ASY1-5D (**Supplementary Table 3**). The predicted domains and immunoprecipitation experiments (Tock et al., 2021) indicate that ASY1 binds to DNA and chromatin at the chromosome axis during wheat meiosis.

### Wheat *asy1* TILLING mutants

*T. turgidum* ‘Kronos’ and *T. aestivum* ‘Cadenza’ *asy1* mutants were identified in the wheat TILLING populations (Krasileva et al., 2017; Appels et al., 2018). Two Kronos lines possessing a premature STOP codon and one line containing a mutation at a splice donor site that retained an intron and subsequent STOP codon were sequenced and verified. The mutations are predicted to truncate and create non-functional ASY1 proteins (*Ttasy1b-1*, 785 C > T, Q 307 > STOP; *Ttasy1b-2*, 148 G > A, W 156 > STOP; *Ttasy1a*, 714 G > A, V 231 > STOP) (**Figure 1** and **Supplementary Figures 2–4**). In Cadenza, only one *asy1* mutant on chromosome 5B was identified, and this is predicted to disrupt the protein function due to a splice donor site mutation and intron retention that led to a STOP codon ((*Taasy1b* (C0971), 1195 G > A, P 254 > STOP)) (**Figure 1** and **Supplementary Figure 5**). The *asy1* transcripts were sequenced from the TILLING lines to confirm that the mutations in the genomic DNA led to stop codons in the coding sequences (**Supplementary Figures 2–5**). As tetraploid Kronos contains four *ASY1* copies, a phenotypic analysis could be performed on hypomorphic mutants: *Ttasy1a* (aaBB), *Ttasy1b-1* (AAbb), *Ttasy1b-2* (AAbb), *Ttasy1Ab* (Aabb), *Ttasy1aB* (aabB), *Ttasy1_1* (aabb), and Cadenza *Taasy1b* (AAbbDD). Seed-set per plant significantly decreased from 22 ± 3 SD per plant in wild-type Kronos (n = 10) to 16 ± 4 SD in *Ttasy1a* (n = 10), 15 ± 3 SD (n = 10) in *Ttasy1a/b*, 8 ± 1 SD in *Ttasy1Ab/aB* (n = 10), and 0 in *Ttasy1_1* (n = 10) as well as from 41 ± 1 SD in wild-type Cadenza (n = 10) to 35 ± 3 SD (n = 10) in *Taasy1b* (*p* < 0.001 Mann–Whitney) (**Supplementary Table 4**). Since fertility is affected and ASY1 is a known meiosis gene, this suggests that meiosis may be disturbed in the mutants leading to infertile gametes. We therefore analyzed the meiotic behavior of these mutants.

### Meiotic progression is delayed in *asy1* hypomorphic mutants

**Figure 2:**
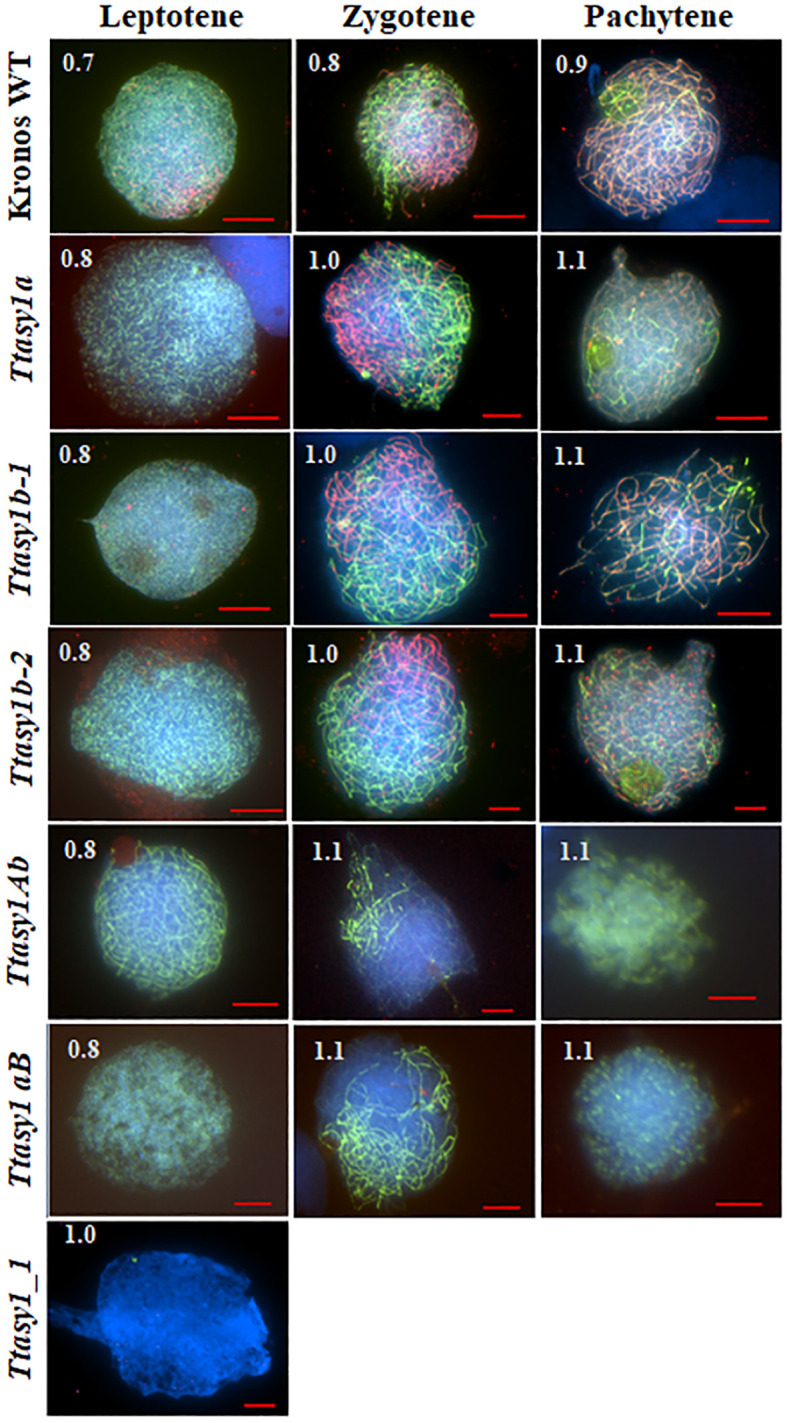
Meiotic prophase I progression in *asy1* mutants. Chromosome axes were marked with ASY1 (green), the synaptonemal complex was marked with ZYP1 (red), and chromosomal DNA was counterstained with DAPI (blue). Anther lengths (mm) were measured for each genotype and prophase 1 stage as shown in the top left corner for each image. Scale bar = 10 µm (Kronos wild type, *Ttasy1Ab*, and *Ttasy1aB*) and 20 µm (*Ttasy1a*, *Ttasy1b-2*, and *Ttasy1_1*).

Meiotic stages in wheat are relatively synchronous and correlate with anther length (Shunmugam et al., 2018). Immunolocalization of ASY1 and ZYP1 was performed on pollen mother cells from wild type and *asy1* hypomorphic mutants to determine if meiotic prophase I progression was affected (**Figure 2**). In the wild type, ASY1 forms linear stretches along the chromosome axes at leptotene, and ZYP1 forms axis-associated foci in anthers 0.7 mm in length (85% nuclei, n = 240) (**Figure 2** and **Supplementary Figure 6**) as previously reported (Sepsi et al., 2017; Osman et al., 2021). At zygotene, ASY1 becomes depleted along the chromosome axes concomitant with ZYP1 polymerization in anthers 0.8 mm in length (90% nuclei, n = 240) (**Figure 2** and **Supplementary Figure 6**) until pachytene when ASY1 is present as a weak, diffuse signal in anthers 0.9 mm in length (90% nuclei, n = 240) (**Figure 2** and **Supplementary Figure 6**). In the *Ttasy1a* and *Ttasy1b* mutants, the ASY1 signal appears indistinguishable compared to the wild type at leptotene, although protein quantities were not determined (**Figure 2**). However, *Ttasy1a* and *Ttasy1b* leptotene nuclei were only observed in 0.8-mm anthers (93%, n = 720, χ^2^ test, *p* < 0.05, **Supplementary Table 5**), compared to 0.7 mm in wild type (85%, n = 240, χ^2^ test, *p* < 0.05, **Supplementary Table 5**), suggesting that prophase I progression was delayed. In the wild type, ZYP1 localized as foci or short stretches in 0.7-mm anthers, but equivalent stages were only observed in anthers 0.8 mm in length in *Ttasy1a* and *Ttasy1b* (**Figure S6**). In the minimum *ASY1* dose mutants (*Ttasy1aB* and *Ttasy1Ab*), leptotene stages were detected in anthers 0.9 mm (95%, n = 480, χ^2^ test, *p* < 0.05, **Figure S6**), indicating a greater delay than the single *Ttasy1a* and *Ttasy1b* mutants (**Figure 2**). Short stretches of ZYP1 were detected in 1.1-mm anthers (n = 480), and in 50% of cells, ZYP1 failed to polymerize, instead forming polycomplexes (**Figure 2**). Neither ASY1 nor ZYP1 was detected on meiotic chromosomes in 0.7–1.1-mm anthers in *Ttasy1_1* (n = 480), suggesting that it was a null asynaptic mutant.

ASY1 labeling at leptotene in Cadenza *Taasy1b* appeared indistinguishable from the wild type, although protein levels were not determined. The A and D copies are expected to be fully functional, although the Set3 PhD finger H3K4me3 domain was not detected in *Ta*ASY1-5D, and this could have a detrimental effect (**Figure S6**). At zygotene, ZYP1 installation in *Taasy1b* occurred as in the wild type, but polymerization was discontinuous and temporally compromised (1.0 mm in 65% nuclei, n = 240 versus 0.8 mm in 85% wild-type nuclei, n = 240), indicating that a reduced dose of *ASY1* delayed meiotic progression in hexaploid wheat (**Figure S6**).

### Correct dosage of ASY1 is required for crossover assurance

**Figure 3:**
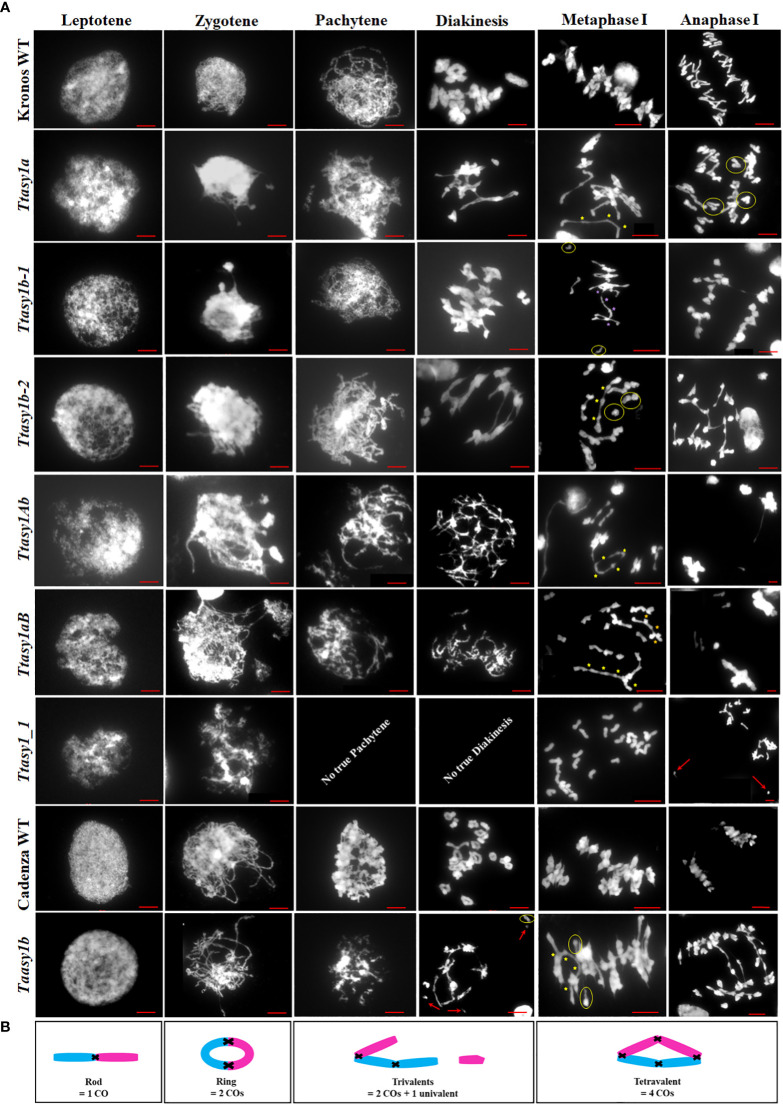
Cytological atlas of *asy1* mutants. **(A)** DAPI-stained meiotic stages from leptotene to anaphase I illustrating phenotypic effects of the *asy1* hypomorphic mutants. Yellow circles highlight univalents, yellow stars indicate chiasmata in multivalents, and red arrows highlight lagging chromosomes. Scale bar = 10 µm. **(B)** Cartoon of chiasma configuration of wheat. The panel depicts bivalent shapes (ring and rod) and trivalent and tetravalent configurations at metaphase I, including points of chiasmata (black crosses) along the chromosomes (blue and pink).

**Figure 4:**
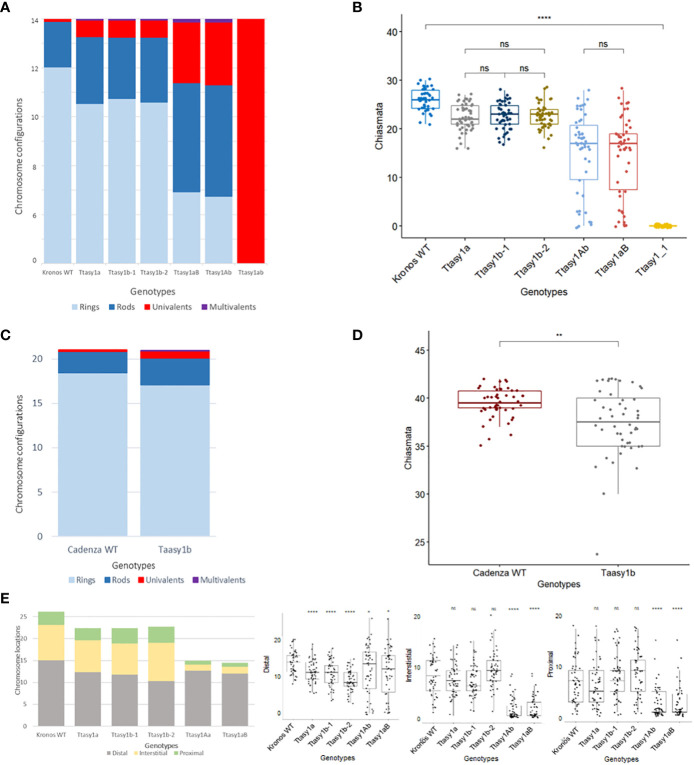
Hypomorphic *asy1* mutants display dosage-dependent reduction in chiasma frequency. Bar charts illustrate the mean values of ring bivalents (sky blue), rod bivalents (dark blue), univalents (red), and multivalents (purple) per cell among **(A)** Kronos wild type and *Ttasy1* mutants and **(C)** Cadenza wild type and *Taasy1b* mutant. Legend is at the bottom. **(B)** Box plots exemplify chiasma frequency per male meiocyte among Kronos wild type and *Ttasy1* mutants. Significant differences are indicated by pairwise Wilcoxon rank sum test (signif. codes: 0 "****" 0.0001 “***” 0.001 “**” 0.01 “*” 0.1 “ns”). The adjustment methods include the Bonferroni correction. **(D)** Box plots exemplify chiasma frequency per male meiocyte between Cadenza wild type and *Taasy1b* mutants. Results of the two-sample t-test are shown (p < 0.001). **(E)** Position of chiasmata (distal, interstitial, proximal) along the chromosomes in *Ttasy1* mutants. Legend is at the bottom. Results of the two-sample t-test are shown (p < 0.001). Significant differences are indicated by pairwise Wilcoxon rank sum test (signif. codes: 0 "****" 0.0001 “***” 0.001 “**” 0.01 “*” 0.1 “ns”).

A cytological analysis was performed on the wild type and *asy1* Kronos mutants with DAPI-stained metaphase I chromosome spreads. Chiasmata ranged from 21 to 30 per nucleus in wild-type Kronos, with a mean of 26 ± 2.2 (n = 50), and each of the 14 pairs of chromosomes received at least one chiasma (**Figure 3A**). In *Ttasy1a*, chiasmata ranged from 16 to 27 per nucleus with a significantly lower mean (22 ± 2.8, n = 50) (pairwise Wilcoxon rank sum test, *p* < 0.01) (**Table 1**) compared to the wild type. Similarly, *Ttasy1b-1* (n = 50) and *Ttasy1b-2* (n = 50) exhibited a mean of 22 ± 2.8 and 23 ± 2.4, respectively (**Figures 3A**, **4A, 4B** and **Table 1**). Chiasma frequency for the *Ttasy1a/b* lines was not significantly different from each other (pairwise Wilcoxon rank sum test, *p* adj > 0.05, *Ttasy1a*, n = 50; *Ttasy1b-1*, n = 50; *Ttasy1b-2*, n = 50), indicating that both A and B sub-genomes provide a similar, non-redundant contribution of ASY1 (**Supplementary Tables 6–12**). Chiasmata were significantly reduced in *Ttasy1Ab* (15.0, n = 50) and *Ttasy1aB* (14.4, n = 50), and no chiasmata were observed in the null mutant *Ttasy1_1* (n = 50) (**Figure 3A**). Similarly, chiasmata were significantly reduced in hexaploid wheat from 39 ± 1.6 (n = 50) in wild type to 37 ± 3.5 (n = 50) in *Taasy1b* (two-sample *t*-test, *p* < 0.001, n = 50 *Taasy1b* and n = 50 Cadenza wild type) (**Figure 4C, D**, **Table 1**, and **Supplementary Table 13**).

**Table 1:** Chiasma frequency and distribution in wheat *asy1* mutants.

Genotypes	Distal chiasma			Interstitial chiasma			Proximal chiasma			Total
	Mean	SD	%	Mean	SD	%	Mean	SD	%	
Kronos WT	15.04	3.34	57.5	8.08	3.74	30.9	3.02	2.02	11.6	26.1
*Ttasy1a*	12.32	3.37	54.9	7.28	2.85	32.4	2.84	2.16	12.7	22.4
*Ttasy1b-1*	11.8	3.75	52.6	7.12	3.2	31.8	3.5	2.22	15.6	22.4
*Ttasy1b-2*	10.32	3.5	45.5	8.7	3.12	38.3	3.68	2.24	16.2	22.7
***p*-Value**	0.00			ns			ns			
*Ttasy1Aa*	12.66	7.44	84.4	1.42	2.2	9.5	0.92	1.48	6.1	15.0
*Ttasy1aB*	12.06	7.26	83.5	1.5	2.16	10.4	0.88	1.48	6.1	14.4
***p*-Value**	0.01			0.00			0.00			

Chiasma frequency at meiotic metaphase I was scored for each genotype, and the mean and standard deviation (SD) are presented. A t-test two-sample distribution was applied to define the statistical significance (p < 0.05).

Chiasma position was also significantly altered in the *asy1* hypomorphic mutants. In the wild type, the majority of chiasmata formed distally to the centromere (57.5%, 15 ± 3), followed by interstitial (31%, 8 ± 4) and proximal (12%, 3 ± 2) (**Figure 4E**). In *Ttasy1a/b*, interstitial and proximal chiasmata were reduced by 1% (n = 150), but in *Ttasy1Ab/aB*, where interstitial and proximal chiasmata decreased from 8 to 1 per nucleus and from 3 to 0.9, respectively, compared to the wild type (n = 100) (**Figure 4E**). In *Ttasy1a/b*, the proportions remained similar, although there was a slight reduction in distal chiasmata (53%, *t*-test, ns) with a minor increase in interstitial (32%, *t*-test, ns) and proximal chiasmata (15%, *t*-test, ns). In *Ttasy1Ab/aB*, distal chiasmata were predominant (84%, *t*-test, *p* adj < 0.001), followed by interstitial (10%, *t*-test, *p* adj < 0.001), and the remaining 6% of chiasmata were proximal (*t*-test, *p* adj < 0.001; **Supplementary Tables 6-12**) (**Figure 4E**). These data indicate that ASY1 is required to create a bias for promoting chiasma formation in the centromere proximal and interstitial regions in wheat.

In all *Ttasy1a/b* and *Ttasy1Ab/aB* hypomorphic mutants, ring bivalents (where at least one chiasma forms in each chromosome arm) were significantly reduced (by 18%, 9 ± 2, n = 150, *t*-test, *p* adj < 0.001; and by 64%, 4 ± 3, n = 100, *t*-test, *p* adj < 0.001, respectively). This was accompanied by a 33% (3.5 ± 2, n = 150, *t*-test, *p* adj < 0.001) increase in rod bivalents (only one chiasma) in *Ttasy1a/b*, which further increased by 1.9-fold (6.5 ± 4.2, n = 100, *t*-test, *p* adj < 0.001) in *Ttasy1Ab/aB* (**Figure 3A**). Lastly, univalents (no chiasma) significantly increased from 0.16 per nucleus in the wild type by fivefold in *Ttasy1a/b* (1 ± 1, n = 150, *t*-test, *p* adj < 0.001) and by 20-fold in *Ttasy1Ab/aB* (3 ± 5, n = 100, *t*-test, *p* adj < 0.001) (**Figure 3A**), revealing loss of the obligate chiasma and an inability to maintain CO assurance. Loss of the obligate chiasma resulted in chromosome mis-segregation and chromosome bridges at anaphase I (**Figure 3A**).

### ASY1 suppresses non-homologous recombination

Ectopic recombination leading to multiple chromosome associations was observed in all hypomorphic *asy1* mutants (**Figures 3A, B**). In *Ttasy1Ab/aB*, multiple chromosome associations per nucleus were observed (0.18 ± 0.5, n = 100). These were classified into three groups of which 44% were tetravalents (4 chromosomes), 33% trivalents (3 chromosomes), and 23% multivalents (more than 4 chromosomes). Multiple chromosome associations were twofold more frequent in *Ttasy1Ab/aB* than in *Ttasy1a/b* (0.08 ± 0.3, n = 150), of which 100% were tetravalents (n = 150). A meiotic cytological analysis was also performed on the hexaploid wheat at metaphase I, revealing an increase in multivalents from 0 in the wild type to 0.06 ± 0.2 (n = 100) in the *Taasy1b* mutant (*p* < 0.005) (**Table 2**).

**Table 2:** Chromosome associations in wheat *asy1* mutants.

Genotypes	Univalent pairs	Bivalent (rod)	Bivalent (ring)	Chiasmata	Multivalents
	Mean ± SD	Mean ± SD	Mean ± SD	Mean ± SD	Mean ± SD
Kronos WT	0.16 ± 0.37	2.6 ± 1.67	11.24 ± 1.66	26.14 ± 2.18	0
*Ttasy1a*	0.94 ± 0.1	3.82 ± 2.26	9.12 ± 2.45	22.44 ± 2.79	0.08 ± 0.27
*Ttasy1b-1*	0.98 ± 1.02	3.50 ± 2.14	9.42 ± 2.29	22.48 ± 2.80	0.06 ± 0.24
*Ttasy1b-2*	0.80 ± 0.81	3.74 ± 1.59	9.28 ± 1.90	22.58 ± 2.44	0.08 ± 0.27
*Ttasy1Ab*	3.44 ± 5.26	6.18 ± 4.29	4.04 ± 3.71	15.14 ± 8.40	0.18 ± 0.48
*Ttasy1aB*	3.56 ± 5.27	6.30 ± 4.22	3.80 ± 3.57	14.58 ± 8.16	0.18 ± 0.48
*Ttasy1_1*	14 ± 0	0	0	0	0
***p*-Value**	0.00	0.00	0.00	0.00	0.00
Cadenza WT	0.14 ± 0.40	2.44 ± 1.43	18.42 ± 1.72	39.48 ± 1.55	0
*Taasy1b*	0.82 ± 0.94	3.02 ± 2.24	17.08 ± 2.80	34.24 ± 10.74	0.06 ± 0.24
***p*-Value**	0.00	0.00	0.00	0.00	0.00

Chromosome configurations at meiotic metaphase I were scored for each genotype, and the mean and standard deviation (SD) are presented. A t-test two-sample distribution was applied to define the statistical significance (p < 0.05).

**Figure 5:**
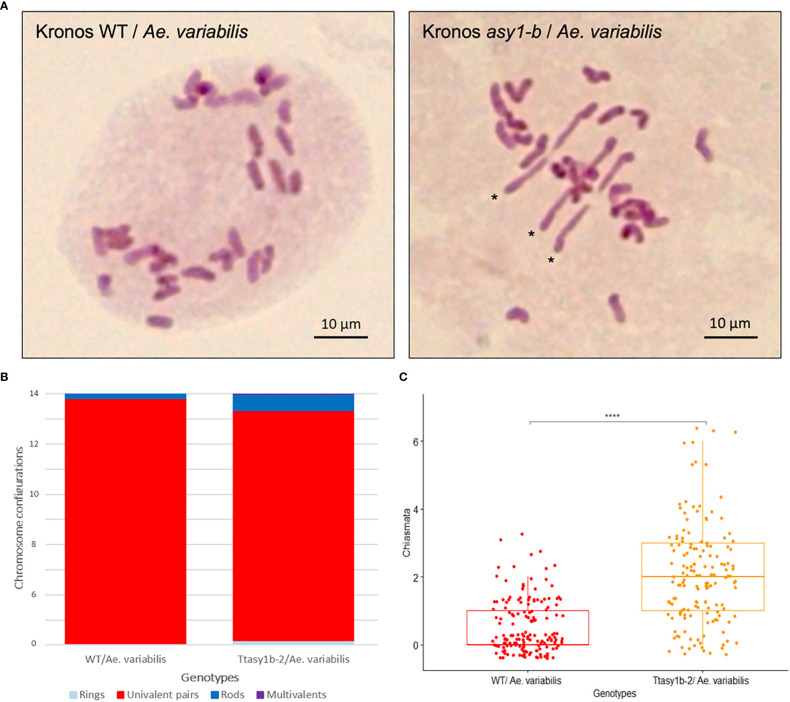
*Ttasy1b-2/Aegilops variabilis* exhibits increased chiasma frequency at metaphase I. **(A)** Metaphase I spread of Kronos wild type/*Ae. variabilis* showing 28 univalents and *Ttasy1b-2/Ae. variabilis* mutants forming three non-homologous bivalents. Asterisks indicate rod bivalents. Scale bar = 10 µm. **(B)** Bar chart illustrates the mean values of ring bivalents (sky blue), rod bivalents (dark blue), univalents (red), and multivalents (purple) per cell among Kronos wild type/*Ae. variabilis* and *Ttasy1b-2*/*Ae. variabilis* mutants. Legend is at the bottom. **(C)** Box plot exemplifies chiasma frequency per male meiocyte among Kronos wild type/*Ae. variabilis* and *Ttasy1b-2/Ae. variabilis* mutants. A two-sample *t*-test was applied to define the statistical significance between wild type*/Ae. variabilis* and *Ttasy1b-2/Ae. variabilis* mutant (signif. codes: 0 “****”).

To determine if the ectopic recombination observed in Kronos and Cadenza *asy1* hypomorphic mutants extended to more divergent genotypes, crosses were made between *Ttasy1b-2* and wheat allotetraploid wild-relative *Ae. variabilis*. Fourteen bivalents would be expected if Kronos and *Ae. variabilis* were capable of forming the obligate chiasma, and 28 univalents would be expected if chiasmata did not form (**Figure 5A**). In the Kronos wild type/*Ae. variabilis* cross, univalents ranged from 22 to 28 with a mean of 26.93 ± 0.12, and bivalents ranged from 0 to 3 with a mean of 0.54 ± 0.06 (n = 155) (**Table 3**), indicating a low level of CO formation between these divergent wheat genotypes. However, in *Ttasy1b-2*/*Ae. variabilis*, the number of univalents decreased in range (16–28, n = 155), with a 3.44-fold increase in the number of bivalents to 1.86 ± 0.1 (*p* < 0.001 Mann–Whitney) (**Figures 5A–C**). The mean chiasma frequency significantly increased in *Ttasy1b-2*/*Ae. variabilis* by 3.75-fold from 0.55 (n = 155) to 2.06 (n = 152) chiasmata per nucleus with a range from 0 to 7 (*p* < 0.001 Mann–Whitney) (**Figures 5B, C**, **Table 3** and **Supplementary Tables 14–18**). This suggests that ASY1 suppresses CO formation between divergent chromosomes in a gene dosage-dependent manner.

**Table 3:** Chiasma counts of Kronos wild type/*Aegilops variabilis* and *Ttasy1b-2*/*Ae. variabilis* mutants.

Genotypes	Anther	Univalents (mean ± SEM)	Bivalents (rod) (mean ± SEM)	Bivalents (ring) (mean ± SEM)	Multivalents (mean ± SEM)	Chiasma frequency (mean ± SEM)	Mean	Fold increased
Kronos WT/*Ae. variabilis*	1 (n = 50)	26.96 ± 0.20	0.52 ± 0.10	0	0	0.52 ± 0.10	0.55	
		(22–28)	(0–3)			(0–3)		
	2 (n = 50)	26.80 ± 0.21	0.56 ± 0,10	0.04 ± 0.03	0	0.64 ± 0.12		
		(22–28)	(0–3)	(0–1)		(0–3)		
	3 (n = 55)	27.02 ± 0.19	0.49 ± 0.10	0	0	0.49 ± 0.10		
		(24–28)	(0–2)			(0–2)		
*Ttasy1b-2*/*Ae. variabilis*	1 (n = 51)	23.92 ± 0.35	1.86 ± 0.17	0.18 ± 0.07	0	2.22 ± 0.20	2.06	3.75
		(18–28)	(0–5)	(0–2)		(0–6)		
	2 (n = 50)	24.26 ± 0.40	1.58 ± 0.19	0.26 ± 0.07	0.02 ± 0.02	2.18 ± 0.25		
		(16–28)	(0–6)	(0–2)	(0–1)	(0–7)		
	3 (n = 51)	24.57 ± 0.32	1.65 ± 0.16	0.04 ± 0.03	0.02 ± 0.02	1.78 ± 0.17		
		(18–28)	(0–5)	(0–1)	(0–1)	(0–6)		
***p*-Value**	0.00	0.00	0.00	ns	ns	0.00	0.00	

Chromosome configurations were scored for each cell in individual anthers for each genotype, and the mean, standard error of the mean (SEM), and range are presented. A t-test two-sample distribution was applied to define the statistical significance (p < 0.05) between WT/Kronos/Ae. variabilis and Ttasy1b-2/Ae. variabilis.

## Discussion

### ASY1 maintains CO assurance and promotes CO formation away from the chromosome ends

Chiasmata were reduced concomitantly with gene dosage in the Kronos hypomorphic *asy1* mutants (WT AABB = 26 chiasmata/cell; AAbb/aaBB =22 chiasmata/cell; Aabb/aaBb = 15 chiasmata/cell; and the null mutant aabb = 0 chiasmata/cell). The *asy1* null mutant phenotype is consistent with the ASY1 rice ortholog *pair1* mutant where only univalents were observed (Nonomura et al., 2004). Kronos possesses 14 pairs of chromosomes, so the mean number of chiasmata in the hypomorphic mutants is sufficient to ensure the obligate CO, although this is not maintained due to the range around the mean, and also, chiasmata are not equally distributed between the chromosomes. We were unable to determine if this was stochastic or that certain chromosomes were more likely to be affected due to the unreliability of oligonucleotide fluorescence *in situ* hybridization (FISH) probes to barcode the chromosomes (data not shown).

The cytological data reveal that wheat ASY1 promotes chiasma formation proximal to the centromeres and along the chromosome arms. This is remarkably similar to previous reports in *A. thaliana* where ASY1 promotes recombination away from the telomeres in a dosage-dependent manner and is essential for the obligate CO (Sanchez-Moran et al., 2007; Lambing et al., 2020; Pochon et al., 2022). In *Arabidopsis*, barley, and wheat, telomeres cluster during leptotene (Armstrong et al., 2001; Higgins et al., 2012; Sepsi et al., 2017), thus providing an early opportunity for nascent strand invasion events in the sub-telomeric regions to precede those in interstitial regions. These early contacts are likely to bias recombination maturation in the distal regions and prevent further COs from forming in the interstitial regions by CO interference (Higgins et al., 2014). Therefore, ASY1 could alleviate this early bias by forming axial bridges between chromosomes at greater distances to promote strand invasion, thereby enabling CO formation away from the chromosome ends.

### Synapsis is dependent on ASY1

ASY1 protein levels were not quantified, but the ASY1 axis signal by immunofluorescence did not appear different in the hypomorphic mutants when compared to the wild type. However, a significant delay in prophase I progression in the hypomorphic mutants associated with a reduction in chiasmata may reflect a lower rate of ASY1 protein production that eventually reached wild-type levels. As no ASY1 protein was detected on the axes in the null *asy1* mutant, it is unlikely that truncated forms of the ASY1 proteins would influence the phenotype in these mutants as dominant negatives. The delay in ZYP1 loading at zygotene is also associated with reduced *ASY1* dosage in the hypomorphic mutants. Incomplete ZYP1 polymerization in *Ttasy1a/b* and its total absence in *Ttasy1Ab/aB* and *Ttasy1_1* led to asynchronous meiotic progression that arrested at pachytene and diplotene. In barley, ZYP1 is required for ~85% COs (Barakate et al., 2014), so a delay in synapsis may have had an additive effect in the wheat *asy1* mutants on chiasma formation as well as loss of function of ASY1 in promoting interhomolog recombination.

### How does ASY1 promote and suppress COs in wheat?

A role for ASY1 in preventing ectopic recombination during meiosis was previously reported (Boden et al., 2009), which is supported by our data. In addition, wheat ASY1 also promotes recombination along the chromosome arms to assuage the telomere-led bias, phenotypically similar to ASY1 in *Arabidopsis*. Therefore, how does ASY1 promote and suppress COs in wheat? ASY1 may be required to provide a minimum number of interhomolog axial bridge contact points between chromosomes in the pairing process, thereby ensuring accurate fidelity so that the homologous chromosomes can synapse and recombine. It is possible that stronger associations would form between homologous chromosomes rather than homoeologous chromosomes due to the stringency of base pairing and hydrogen bonding of the single-end invasions, promoted by ASY1 and DMC1. If the minimum number of contact points is reduced below a threshold, such as what could happen in the *asy1* hypomorphic mutants, then the fidelity of chromosome recognition may be impaired leading to ectopic recombination in the tetraploid/hexaploid mutants and increased chiasmata in *asy1*/*Ae. variabilis*. The delay in meiotic progression in the *asy1* hypomorphic mutants may reflect a surveillance system performing sub-optimally that is required to monitor accurate pairing and ensure that synapsis initiates between homologous, rather than homoeologous chromosomes.

### Does *ASY1* share the characteristics of pairing homoeologous loci in wheat?

The pairing homoeologous (*Ph*) loci in wheat negatively act on recombination between chromosomes of diverged species. Here, we show that the mean chiasma frequency increased by 3.75-fold in Kronos *Ttasy1b-2*/*Ae. variabilis* when compared to wild type/*Ae. variabilis*, indicating that ASY1 suppresses recombination between divergent chromosomes. This is similar to the *Ph* loci, although to a lesser extent as chiasmata increased by 8.3-fold/cell in *Ph1* (*zip4 5B*) and up to 5.5-fold/cell in *Ph2* (*msh7-3D*) hexaploid wheat mutants when crossed with *Ae. variabilis. TaASY1-5B* is located on the long arm of chromosome 5, separated by 33.5 Mb of DNA from the class I CO gene *ZIP4-5B* (Rey et al., 2017; Martín et al., 2018). The novel duplication of *ZIP4* on chromosome 5B is indicative of adaptive evolution, whereas there are no obvious hallmarks of adaptation at the *ASY1* 5B locus (although this requires further investigation). However, phenotypic similarities exist between *Ph1* and *asy1* such as an increase in homoeologous recombination and a delay in synapsis. Pochon et al. (2022) reported that not all MLH1 foci maturate into COs in *Arabidopsis asy1* mutants, reminiscent of the *Ph1* phenotype, suggesting a possible association between *asy1* and *Ph1* (Martín et al., 2014; Martín et al., 2017; Pochon et al., 2022). Moreover, in *ph1b*, localization of ASY1 was perturbed, adopting a spiral-like pattern during zygotene and pachytene (Boden et al., 2009). Surprisingly, no multivalents were observed in *Tazip4-B2* mutant lines, whereas they are observed in *ph1b* at a low frequency (trivalents 0.2% and tetravalents 0.37%) (Rey et al., 2017) and in the *Taasy1* 5B mutant line.

The chromosome axis has been implicated in adaptation to meiotic recombination in autotetraploid *Arabidopsis arenosa* and *Arabidopsis lyrata*. *ASY1* and *ASY3* alleles are under selection in these tetraploids that distalize chiasmata to the chromosome ends and reduce their number (Morgan et al., 2020; Seear et al., 2020). This implicates *ASY1* as a major gene required to stabilize both allopolyploid and autopolyploid meiotic recombination. It also raises the potential to combine *zip4 5B* and *asy1* (*5A*, *5B*, or *5D*) to increase introgression from wheat wild relatives.

In conclusion, this study provides further support for the role of *ASY1* in controlling CO number and position as well as CO assurance in plants. The dosage sensitivity of *ASY1* in wheat is similar to the haplo-insufficiency reported in *Arabidopsis* (Lambing et al., 2020), suggesting that ASY1 performs a conserved role in both diploid and polyploid species. The fidelity of accurate chromosome pairing is reduced in the hypomorphic *asy1* wheat mutants, leading to ectopic recombination This indicates that ASY1 plays a major role in chromosome recognition and may bias recombination toward the homolog rather than homoeologous chromosomes by monitoring DNA sequence homology during stable strand invasion. Thus, wheat hypomorphic *asy1* mutants could provide a tool to enhance the introgression of agronomically important traits from wheat wild relatives into elite varieties.

## Data availability statement

The original contributions presented in the study are included in the article/**Supplementary Material**. Further inquiries can be directed to the corresponding author.

## Author contributions

JH and PS designed the research performed by CD and HS. JH, PS, CD, and HS analyzed the data and wrote the manuscript. All authors contributed to the article and approved the submitted version.
