# Supplementary material for: ASYNAPSIS 1 ensures crossover fidelity in polyploid wheat by promoting homologous recombination and suppressing non-homologous recombination
Source: Front Plant Sci. 2023 May 22;14:1188347. doi: 10.3389/fpls.2023.1188347 (PMC10239940; doi:10.3389/fpls.2023.1188347)
Supplement: Supplementary file 1 [file DataSheet_1.docx]

**Supplementary materials**

>Kronos ASY1-5A

ATGCAGATGATGGCTCAGAAGACGAAGGAGGCGGAGATCACGGAGCAGGACTCGCTGCTTCTAACAAGGAATTTGCTCCGGATTGCTATATACAACATCAGCTACATCAGAGGCCTATTCCCTGAGAAGTACTTCAATGATAAGTCTGTTCCGGCACTAGAGATGAAGATTAAGAAGCTGATGCCCATGGATGCTGAATCCAGGAGGTTGATTGATTGGATGGAGAAAGGTGTCTATGATGCCTTACAAAAGAAATATCTCAAGACCCTTCTCTTCTGTATATGTGAGAAGGAGGAAGGCCCAATGATTGAAGAGTATGCCTTCTCATTTAGCTACCCCAACACAAACGGGGAGGAAGTTGCAATGAACATGAGTCGCACAGGGAGCAAAAAGAATAGTGCCACATTCAAGTCAAATGCAGCAGAAGTCACTCCTGATCAGATGAGGAGCTCTGCTTGTAAGATGATCAGAACGCTAGTTTCACTTATGAGGACCTTGGACCAAATGCCAGAGGAGCGAACCATTCTAATGAAGCTGCTATACTATGATGATGCTACACCTGAGGATTACGAGCCTCCCTTCTTTAAGGGTTGTGCTGAGAATGAGGCCGTAAATATATGGAACAAGAACCCCTTGAAGATGGAAGTGGGGAATGTCAATAGCAAGCATCTTGTGTTAGCTTTGAAGGTTAAGAGTGTCCTTGATCCATGTGATGCTAATGATGCTAACAGTGATGATGACAAGATGAGCGTGGGTCATGAGTCAGACCAAGATGACTTTACGGACACCGAGGTTCACCCATCTGAAGTGGATCGTTACGTCATTGCTCCTAATGATGGTAATGGCAAAGGTCAAAGTGGTACAAACTCAGATGATGAAACTCAAGATGCTGCTCATGAGGAAGAGCTAACAGCTCAAGTAAGAGCATGGATATGCTCAAGAAACATGGGTACTGTTAATGCTTCAGATGTCCTTTCCAACTACCCTGACATATCATTGGTAATTCTTGCAGATATTTTGGAGAGGCTACTTAAAGATGGTTTACTTTCGAGGGCAGGCAAGGATGGTTATGCTGTTAACAAGATTACTGATCCCAAAACACCCTACATAAAGGAAGAGGTTGCCATGCACAATGTTTCACCTACTGAAGGAACCAAGAACAACAGTGGAGATCTGATGTATATGAAGGCATTATACCACGCACTTCCAATGGATTATGTGACTATAGCTAAGCTTCAGGGCAAGCTTGATGGCGAAGCCAACCAGAGCACAGTCCGAAAGTTGATGGACAAAATGGTGCAAGATGGATACATTAAGAATTCAGGCAACAGAAGATTAGGCAAAGCTGTCATTCACTCTGAAGTCACCAACAGAAAGCTCCTTGAGATAAAGAAGATACTGGAAGTTGATATCACTGAAGACATGGCAATTGATACCAACGCAAGGCCTGCTGAGTTTGATCGCAGAGATCACCAAACGGCTGACCAGGAAATGAAAGATGGCTCGACAAACGGCCGCTTCCAGTCAGTTGGATCTGATCTTACCCGCACACGGGAGCTGCCGGAGCAGCAGCAGACTAACAAGGACCCAAGCAGGACTCCCACAAGCAATCGCGAGTCGGCCACGTCCCTGGAGAGTGGGGTGCTCGGGCAGAGGATCAGGAAGTCTCTGGCTGGCGAAGAGTCGATGTGCATGCCGGACAAGCGGACCAGGAAGGCCAGCATGGTGAAGGAGCCGATCCTCCAGCAGGTCAAGCGCCAGAAGTCCTAG

>Kronos ASY1-5B

ATGGCTCAGAAGACGAAGGAGGCGGAGATCACGGAGCAGGACTCGCTGCTTCTAACAAGGAATTTGCTCCGGATTGCTATATACAACATCAGCTACATCAGAGGCCTATTCCCTGAGAAGTACTTCAATGATAAGTCTGTTCCAGCACTAGAGATGAAGATTAAGAAGCTGATGCCCATGGATGCTGAATCCAGGAGGTTGATTGATTGGATGGAGAAAGGTGTCTATGATGCCTTACAAAAGAAATATCTCAAGACCCTTCTCTTCTGTATATGTGAGAAGGAGGAAGGCCCCATGATTGAAGAGTATGCCTTCTCATTTAGCTACCCCAACACAAACGGGGAAGAAGTTGCAATGAACATGAGTCGCACAGGGAGCAAAAAGAATAGTGCCACATTCAAGTCAAATGCAGCAGAAGTCACTCCTGACCAGATGAGGAGCTCTGCTTGTAAGATGATCAGAACGCTGGTTTCACTTATGAGGACCTTGGATCAAATGCCAGAGGAGCGAACCATTCTGATGAAGCTGCTATACTATGATGATGCCACACCTGAGGATTACGAGCCTCCCTTCTTTAAGGGTTGTGCTGAGAATGAAGCCGTAAATATATGGAACAAGAACCCCTTGAAGATGGAAGTGGGGAATGTCAATAGCAAGCATCTTGTGTTAGCTTTGAAGGTTAAGAGTGTCCTTGATCCATGTGATGCTAATGATGCTAACAGTGATGATGACAAGATGAGCTTGGGTCGTGAGTCAGACCAAGATAATGATTTATCGGACACCGAGGTTCGCCCATCTGAAGTGGATCGTTACGTCGTTGCTCCTAATGATGGAAATTGCAAAGGTCAGAGTGGTACAAACTCAGAAGATGAAACTCAAGATGCTGCTCATGAGGAAGAGCTAACAGCTCAAGTAAGAGCATGGATATGCTCAAGAGACATGGGTAGTGTTAATGCTTCAGATGTCCTTTCCAACTACCCTGACATATCATTGGAAATGGTGGAAGATATTTTGGAGAGGCTACTTAAAGATGGTTTACTTTCCAGGGCAGGCAAGGATGGTTATGCTGTTAACAAGGTTACTGATCCCAAAACACCCTACATAAAGAAAGAGAAAGAGGTTGCCATGCACAATGTTACACCTACTGAAGGAACCAAAAACAACGACGCAGATGTGATGTACATGAAGGCATTATACCATGCACTTCCAATGGATTATGTGACTGTAGCTAAGCTTCAGAGCAAGCTTGATGGTGAAGCCAACCAGACCACAGTCCGAAAGTTGATGGACAAAATGGTGCAAGATGGATACATTAAGAATTCAGGCAACAGAAGATTAGGAAAAGCTGTCATTCATTCTGAAGTCACCAACAGAAAGCTCCTTGAGATAAAAAAGATACTGGAAGTTGATATCACTGATGAAATGGCAATTGACACCAACGCAAGGCCTGCTGAGTTTGACCGCAGAGATCATCAGATAACTGACCAGGAAATGAAAGATGGCTCGACAAACGGCCGCTTCCAGTCAGTTGGATCTGATCTTACCCGCACACGGGAGCTACCGGAGCAGCAGCAGAATAACAAGGACCCAAGCAGGACTCCCACAAGCAATCGCGAGTCGGCTATGTCCCTGGAGAGTGGGGTGCTCGGGCAGAGGATCAGGAAGTCTCTGGCTGGCGAAGAGTCGATGGGCACGCCGGACAAGCGGACCAGGAAGACCAGCATGGTGAAGGAGCCGATCCTCCAGCAGGTCAAGCGCCAGAAGTCCTAG

>Cadenza ASY1-5A

ATGCAGATGATGGCTCAGAAGACGAAGGAGGCGGAGATCACGGAGCAGGACTCGCTGCTTCTAACAAGGAATTTGCTCCGGATTGCTATATACAACATCAGCTACATCAGAGGCCTATTCCCTGAGAAGTACTTCAATGATAAGTCTGTTCCGGCACTAGAGATGAAGATTAAGAAGCTGATGCCCATGGATGCTGAATCCAGGAGGTTGATTGATTGGATGGAGAAAGGTGTCTATGATGCCTTACAAAAGAAATATCTCAAGACCCTTCTCTTCTGTATATGTGAGAAGGAGGAAGGCCCAATGATTGAAGAGTATGCCTTCTCATTTAGCTACCCCAACACAAACGGGGAGGAAGTTGCAATGAACATGAGTCGCACAGGGAGCAAAAAGAATAGTGCCACATTCAAGTCAAATGCAGCAGAAGTCACTCCTGATCAGATGAGGAGCTCTGCTTGTAAGATGATCAGAACGCTAGTTTCACTTATGAGGACCTTGGACCAAATGCCAGAGGAGCGAACCATTCTAATGAAGCTGCTATACTATGATGATGCTACACCTGAGGATTACGAGCCTCCCTTCTTTAAGGGTTGTGCTGAGAATGAGGCCGTAAATATATGGAACAAGAACCCCTTGAAGATGGAAGTGGGGAATGTCAATAGCAAGCATCTTGTGTTAGCTTTGAAGGTTAAGAGTGTCCTTGATCCATGTGATGCTAATGATGCTAACAGTGATGATGACAAGATGAGCGTGGGTCATGAGTCAGACCAAGATGACTTTACGGACACCGAGGTTCACCCATCTGAAGTGGATCGTTACGTCATTGCTCCTAATGATGGTAATGGCAAAGGTCAAAGTGGTACAAACTCAGATGATGAAACTCAAGATGCTGCTCATGAGGAAGAGCTAACAGCTCAAGTAAGAGCATGGATATGCTCAAGAAACATGGGTACTGTTAATGCTTCAGATGTCCTTTCCAACTACCCTGACATATCATTGGAAATGGTGGAAAAATATTTGGAGAGGCTACTTAAAGATGGTTTACTTTCGAGGGCAGGCAAGGATGGTTATGCTGTTAACAAGATTACTGATCCCAAAACACCCTACATAAAGGAAGAGGTTGCCATGCACAATGTTTCACCTACTGAAGGAACCAAGAACAACAGTGGAGATCTGATGTATATGAAGGCATTATACCACGCACTTCCAATGGATTATGTGACTATAGCTAAGCTTCAGGGCAAGCTTGATGGCGAAGCCAACCAGAGCACAGTCCGAAAGTTGATGGACAAAATGGTGCAAGATGGATACATTAAGAATTCAGGCAACAGAAGATTAGGCAAAGCTGTCATTCACTCTGAAGTCACCAACAGAAAGCTCCTTGAGATAAAGAAGATACTGGAAGTTGATATCACTGAAGACATGGCAATTGATACCAACGCAAGGCCTGCTGAGTTTGATCGCAGAGATCACCAAACGGCTGACCAGGAAATGAAAGATGGCTCGACAAACGGCCGCTTCCAGTCAGTTGGATCTGATCTTACCCGCACACGGGAGCTGCCGGAGCAGCAGCAGACTAACAAGGACCCAAGCAGGACTCCCACAAGCAATCGCGAGTCGGCCACGTCCCTGGAGAGTGGGGTGCTCGGGCAGAGGATCAGGAAGTCTCTGGCTGGCGAAGAGTCGATGTGCATGCCGGACAAGCGGACCAGGAAGGCCAGCATGGTGAAGGAGCCGATCCTCCAGCAGGTCAAGCGCCAGAAGTCCTAG

>Cadenza ASY1-5B

ATGGCTCAGAAGACGAAGGAGGCGGAGATCACGGAGCAGGACTCGCTGCTTCTAACAAGGAATTTGCTCCGGATTGCTATATACAACATCAGCTACATCAGAGGCCTATTCCCTGAGAAGTACTTCAATGATAAGTCTGTTCCAGCACTAGAGATGAAGATTAAGAAGCTGATGCCCATGGATGCTGAATCCAGGAGGTTGATTGATTGGATGGAGAAAGGTGTCTATGATGCCTTACAAAAGAAATATCTCAAGACCCTTCTCTTCTGTATATGTGAGAAGGAGGAAGGCCCCATGATTGAAGAGTATGCCTTCTCATTTAGCTACCCCAACACAAACGGGGAAGAAGTTGCAATGAACATGAGTCGCACAGGGAGCAAAAAGAATAGTGCCACATTCAAGTCAAATGCAGCAGAAGTCACTCCTGACCAGATGAGGAGCTCTGCTTGTAAGATGATCAGAACGCTGGTTTCACTTATGAGGACCTTGGATCAAATGCCAGAGGAGCGAACCATTCTGATGAAGCTGCTATACTATGATGATGCCACACCTGAGGATTACGAGCCTCCCTTCTTTAAGGGTTGTGCTGAGAATGAAGCCGTAAATATATGGAACAAGAACCCCTTGAAGATGGAAGTGGGGAATGTCAATAGCAAGCATCTTGTGTTAGCTTTGAAGGTTAAGAGTGTCCTTGATCCATGTGATGCTAATGATGCTAACAGTGATGATGACAAGATGAGCTTGGGTCGTGAGTCAGACCAAGATAATGATTTATCGGACACCGAGGTTCGCCCATCTGAAGTGGATCGTTACGTCGTTGCTCCTAATGATGGAAATTGCAAAGGTCAGAGTGTTACAAACTCAGAAGATGAAACTCAAGATGCTGCTCATGAGGAAGAGCTAACAGCTCAAGTAAGAGCATGGATATGCTCAAGAGACATGGGTAGTGTTAATGCTTCAGATGTCCTTTCCAACTACCCTGACATATCATTGGAAATGGTGGAAGATATTTTGGAGAGGCTACTTAAAGATGGTTTACTTTCCAGGGCAGGCAAGGATGGTTATGCTGTTAACAAGGTTACTGATCCCAAAACACCCTACATAAAGAAAGAGAAAGAGGTTGCCATGCACAATGTTACACCTACTGAAGGAACCAAAAACAACGACGCAGATGTGATGTACATGAAGGCATTATACCATGCACTTCCAATGGATTATGTGACTGTAGCTAAGCTTCAGAGCAAGCTTGATGGTGAAGCCAACCAGACCACAGTCCGAAAGTTGATGGACAAAATGGTGCAAGATGGATACATTAAGAATTCAGGCAACAGAAGATTAGGAAAAGCTGTCATTCATTCTGAAGTCACCAACAGAAAGCTCCTTGAGATAAAAAAGATACTGGAAGTTGATATCACTGATGAAATGGCAATTGACACCAACGCAAGGCCTGCTGAGTTTGACCGCAGAGATCATCAGATAACTGACCAGGAAATGAAAGATGGCTCGACAAACGGCCGCTTCCAGTCAGTTGGATCTGATCTTACCCGCACACGGGAGCTACCGGAGCAGCAGCAGAATAACAAGGACCCAAGCAGGACTCCCACAAGCAATCGCGAGTCGGCTATGTCCCTGGAGAGTGGGGTGCTCGGGCAGAGGATCAGGAAGTCTCTGGCTGGCGAAGAGTCGATGGGCACGCCGGACAAGCGGACCAGGAAGACCAGCATGGTGAAGGAGCCGATCCTCCAGCAGGTCAAGCGCCAGAAGTCCTAG

>Cadenza ASY1-5D

ATGGCTCAGAAGACGAAGGAGGCGGAGATCACGGAGCAGGACTCGCTGCTTCTAACAAGGAATTTGCTCCGGATTGCTATATACAACATCAGCTACATCAGAGGCCTATTCCCTGAGAAGTACTTCAATGATAAGTCTGTTCCAGCACTAGAGATGAAGATTAAGAAGCTGATGCCCATGGATGCTGAATCCAGGAGGTTGATTGATTGGATGGAGAAAGGTGTTTATGATGCCTTACAAAAGAAATATCTCAAGACCCTTCTCTTCTGTATATGTGAGAAGGAGGAAGGCCCAATGATTGAAGAGTATGCCTTCTCATTTAGCTACCCCAACACAAACGGGGAAGAAGTTGCAATGAACATGAGTCGCACAGGGAGCAAAAAGAATAGTGCCACATTCAAGTCAAATGCAGCAGAAGTCACTCCTGATCAGATGAGGAGCTCTGCTTGTAAGATGATCAGAACGCTGGTTTCACTTATGAGGACCTTGGATCAAATGCCAGAGGAGCGAACCATTCTGATGAAGCTGCTATACTATGATGATGCCACACCTGAGGATTACGAGCCTCCCTTCTTTAAGGGTTGTGCTGAGAATGAGGCTGTAAATATATGGAACAAGAACCCCTTGAAGATGGAAGTGGGGAATGTCAATAGCAAGCATCTTGTGTTAGCTTTGAAGGTTAAGAGTGTCCTTGATCCGTGTGATGCTAATGATGCTAACAGTGATGATGACAAGATGAGCTTGGGTCGTGAGTCAGACCAAGATGATGACTTATCGGACACCGAGGTTCGCCCATCTGAAGTGGATCGTTACGTCGTTGCTCCTAATGATGGAAATTGCAAAGGTCAAAGTGGTACAAACTCAGAAGATGAAACTCAAGATGCTGCTCATGAGGAAGAGCTAACAGCTCAAGTAAGAGCATGGATATGCTCAAGAGACATGGGTACTGTTAGTGCTTCAGATGTCCTTTCCAACTACCCTGACATATCATTGGAAATGGTGGAAGATATTTTGGAGAGGCTACTTAAAGATGGTTTACTTTCCAGGGCAGGCAAGGATGGTTATGCTGTTAACAAGGTTACTGATCCCAAAACACCCTACATAAAGAAAGAGAGAGAGGTTGCCATGCACAATGTTTCACCTACTGAAGGAACCAAAAACAACGATGCAGATCTGATGTACATGAAGGCATTATACCACGCACTTCCAATGGATTATGTGACTATAGCTAAGCTTCAGGGCAAGCTTGATGGCGAAGCCAACCAGAGCACAGTCCGAAAGTTGATGGACAAAATGGTGCAAGATGGATACATTAAGAATTCAGGCAACAGAAGATTAGGAAAAGCTGTCATTCATTCTGAAGTCACCAACAGAAAGCTCCTTGAGATAAAAAAGATACTGGAAGTTGATATCACTGATGAAATGGCAGTTGATACCAATGCAAGGCCTGCTGAGTTTGACCGCAGAGATCATCAGATGGCTGACCAGGAAATGAAAGATGGCTCGACAAACGGCCGCTTCCAGTCAGTTGGATCTGATCTTACCCGCACACGGGACCTACCAGAGCAGCAGCAGAATAACAAGGACCCAAGCAGGACTCCCACAAGCAATCGCGAGTCGGCTACGTCCCTGGAGAGTGGGGTGCTCGGGCAGAGGATCAGGAAGTCTCTGGCTCGCGAAGAGTCGATGTGCACGCCGGACAAGCGGACCAGGAAGACCAGCATGGTGAAGGAGCCGATCCTCCAGCAGGTCAAGCGCCAGAAGTCCTAG

>Kronos ASY1-5A

MQMMAQKTKEAEITEQDSLLLTRNLLRIAIYNISYIRGLFPEKYFNDKSVPALEMKIKKLMPMDAESRRLIDWMEKGVYDALQKKYLKTLLFCICEKEEGPMIEEYAFSFSYPNTNGEEVAMNMSRTGSKKNSATFKSNAAEVTPDQMRSSACKMIRTLVSLMRTLDQMPEERTILMKLLYYDDATPEDYEPPFFKGCAENEAVNIWNKNPLKMEVGNVNSKHLVLALKVKSVLDPCDANDANSDDDKMSVGHESDQDDFTDTEVHPSEVDRYVIAPNDGNGKGQSGTNSDDETQDAAHEEELTAQVRAWICSRNMGTVNASDVLSNYPDISLVILADILERLLKDGLLSRAGKDGYAVNKITDPKTPYIKEEVAMHNVSPTEGTKNNSGDLMYMKALYHALPMDYVTIAKLQGKLDGEANQSTVRKLMDKMVQDGYIKNSGNRRLGKAVIHSEVTNRKLLEIKKILEVDITEDMAIDTNARPAEFDRRDHQTADQEMKDGSTNGRFQSVGSDLTRTRELPEQQQTNKDPSRTPTSNRESATSLESGVLGQRIRKSLAGEESMCMPDKRTRKASMVKEPILQQVKRQKS

>Kronos ASY1-5B

MAQKTKEAEITEQDSLLLTRNLLRIAIYNISYIRGLFPEKYFNDKSVPALEMKIKKLMPMDAESRRLIDWMEKGVYDALQKKYLKTLLFCICEKEEGPMIEEYAFSFSYPNTNGEEVAMNMSRTGSKKNSATFKSNAAEVTPDQMRSSACKMIRTLVSLMRTLDQMPEERTILMKLLYYDDATPEDYEPPFFKGCAENEAVNIWNKNPLKMEVGNVNSKHLVLALKVKSVLDPCDANDANSDDDKMSLGRESDQDNDLSDTEVRPSEVDRYVVAPNDGNCKGQSGTNSEDETQDAAHEEELTAQVRAWICSRDMGSVNASDVLSNYPDISLEMVEDILERLLKDGLLSRAGKDGYAVNKVTDPKTPYIKKEKEVAMHNVTPTEGTKNNDADVMYMKALYHALPMDYVTVAKLQSKLDGEANQTTVRKLMDKMVQDGYIKNSGNRRLGKAVIHSEVTNRKLLEIKKILEVDITDEMAIDTNARPAEFDRRDHQITDQEMKDGSTNGRFQSVGSDLTRTRELPEQQQNNKDPSRTPTSNRESAMSLESGVLGQRIRKSLAGEESMGTPDKRTRKTSMVKEPILQQVKRQKS

>Cadenza ASY1-5A

MQMMAQKTKEAEITEQDSLLLTRNLLRIAIYNISYIRGLFPEKYFNDKSVPALEMKIKKLMPMDAESRRLIDWMEKGVYDALQKKYLKTLLFCICEKEEGPMIEEYAFSFSYPNTNGEEVAMNMSRTGSKKNSATFKSNAAEVTPDQMRSSACKMIRTLVSLMRTLDQMPEERTILMKLLYYDDATPEDYEPPFFKGCAENEAVNIWNKNPLKMEVGNVNSKHLVLALKVKSVLDPCDANDANSDDDKMSVGHESDQDDFTDTEVHPSEVDRYVIAPNDGNGKGQSGTNSDDETQDAAHEEELTAQVRAWICSRNMGTVNASDVLSNYPDISLEMVEKYLERLLKDGLLSRAGKDGYAVNKITDPKTPYIKEEVAMHNVSPTEGTKNNSGDLMYMKALYHALPMDYVTIAKLQGKLDGEANQSTVRKLMDKMVQDGYIKNSGNRRLGKAVIHSEVTNRKLLEIKKILEVDITEDMAIDTNARPAEFDRRDHQTADQEMKDGSTNGRFQSVGSDLTRTRELPEQQQTNKDPSRTPTSNRESATSLESGVLGQRIRKSLAGEESMCMPDKRTRKASMVKEPILQQVKRQKS

>Cadenza ASY1-5B

MAQKTKEAEITEQDSLLLTRNLLRIAIYNISYIRGLFPEKYFNDKSVPALEMKIKKLMPMDAESRRLIDWMEKGVYDALQKKYLKTLLFCICEKEEGPMIEEYAFSFSYPNTNGEEVAMNMSRTGSKKNSATFKSNAAEVTPDQMRSSACKMIRTLVSLMRTLDQMPEERTILMKLLYYDDATPEDYEPPFFKGCAENEAVNIWNKNPLKMEVGNVNSKHLVLALKVKSVLDPCDANDANSDDDKMSLGRESDQDNDLSDTEVRPSEVDRYVVAPNDGNCKGQSVTNSEDETQDAAHEEELTAQVRAWICSRDMGSVNASDVLSNYPDISLEMVEDILERLLKDGLLSRAGKDGYAVNKVTDPKTPYIKKEKEVAMHNVTPTEGTKNNDADVMYMKALYHALPMDYVTVAKLQSKLDGEANQTTVRKLMDKMVQDGYIKNSGNRRLGKAVIHSEVTNRKLLEIKKILEVDITDEMAIDTNARPAEFDRRDHQITDQEMKDGSTNGRFQSVGSDLTRTRELPEQQQNNKDPSRTPTSNRESAMSLESGVLGQRIRKSLAGEESMGTPDKRTRKTSMVKEPILQQVKRQKS

>Cadenza ASY1-5D

MAQKTKEAEITEQDSLLLTRNLLRIAIYNISYIRGLFPEKYFNDKSVPALEMKIKKLMPMDAESRRLIDWMEKGVYDALQKKYLKTLLFCICEKEEGPMIEEYAFSFSYPNTNGEEVAMNMSRTGSKKNSATFKSNAAEVTPDQMRSSACKMIRTLVSLMRTLDQMPEERTILMKLLYYDDATPEDYEPPFFKGCAENEAVNIWNKNPLKMEVGNVNSKHLVLALKVKSVLDPCDANDANSDDDKMSLGRESDQDDDLSDTEVRPSEVDRYVVAPNDGNCKGQSGTNSEDETQDAAHEEELTAQVRAWICSRDMGTVSASDVLSNYPDISLEMVEDILERLLKDGLLSRAGKDGYAVNKVTDPKTPYIKKEREVAMHNVSPTEGTKNNDADLMYMKALYHALPMDYVTIAKLQGKLDGEANQSTVRKLMDKMVQDGYIKNSGNRRLGKAVIHSEVTNRKLLEIKKILEVDITDEMAVDTNARPAEFDRRDHQMADQEMKDGSTNGRFQSVGSDLTRTRDLPEQQQNNKDPSRTPTSNRESATSLESGVLGQRIRKSLAREESMCTPDKRTRKTSMVKEPILQQVKRQKS

>Consensus sequence

MAQKTKEAEITEQDSLLLTRNLLRIAIYNISYIRGLFPEKYFNDKSVPALEMKIKKLMPMDAESRRLIDWMEKGVYDALQKKYLKTLLFCICEKEEGPMIEEYAFSFSYPNTNGEEVAMNMSRTGSKKNSATFKSNAAEVTPDQMRSSACKMIRTLVSLMRTLDQMPEERTILMKLLYYDDATPEDYEPPFFKGCAENEAVNIWNKNPLKMEVGNVNSKHLVLALKVKSVLDPCDANDANSDDDKMSLGRESDQDDDLSDTEVRPSEVDRYVVAPNDGNCKGQSGTNSEDETQDAAHEEELTAQVRAWICSRDMGSVNASDVLSNYPDISLEMVEDILERLLKDGLLSRAGKDGYAVNKVTDPKTPYIKKEKEVAMHNVSPTEGTKNNDADLMYMKALYHALPMDYVTIAKLQGKLDGEANQSTVRKLMDKMVQDGYIKNSGNRRLGKAVIHSEVTNRKLLEIKKILEVDITDEMAIDTNARPAEFDRRDHQIADQEMKDGSTNGRFQSVGSDLTRTRELPEQQQNNKDPSRTPTSNRESATSLESGVLGQRIRKSLAGEESMCTPDKRTRKTSMVKEPILQQVKRQKS

**Supplementary Figure 1. Polyploid wheat ASY1 coding sequences and predicted proteins**


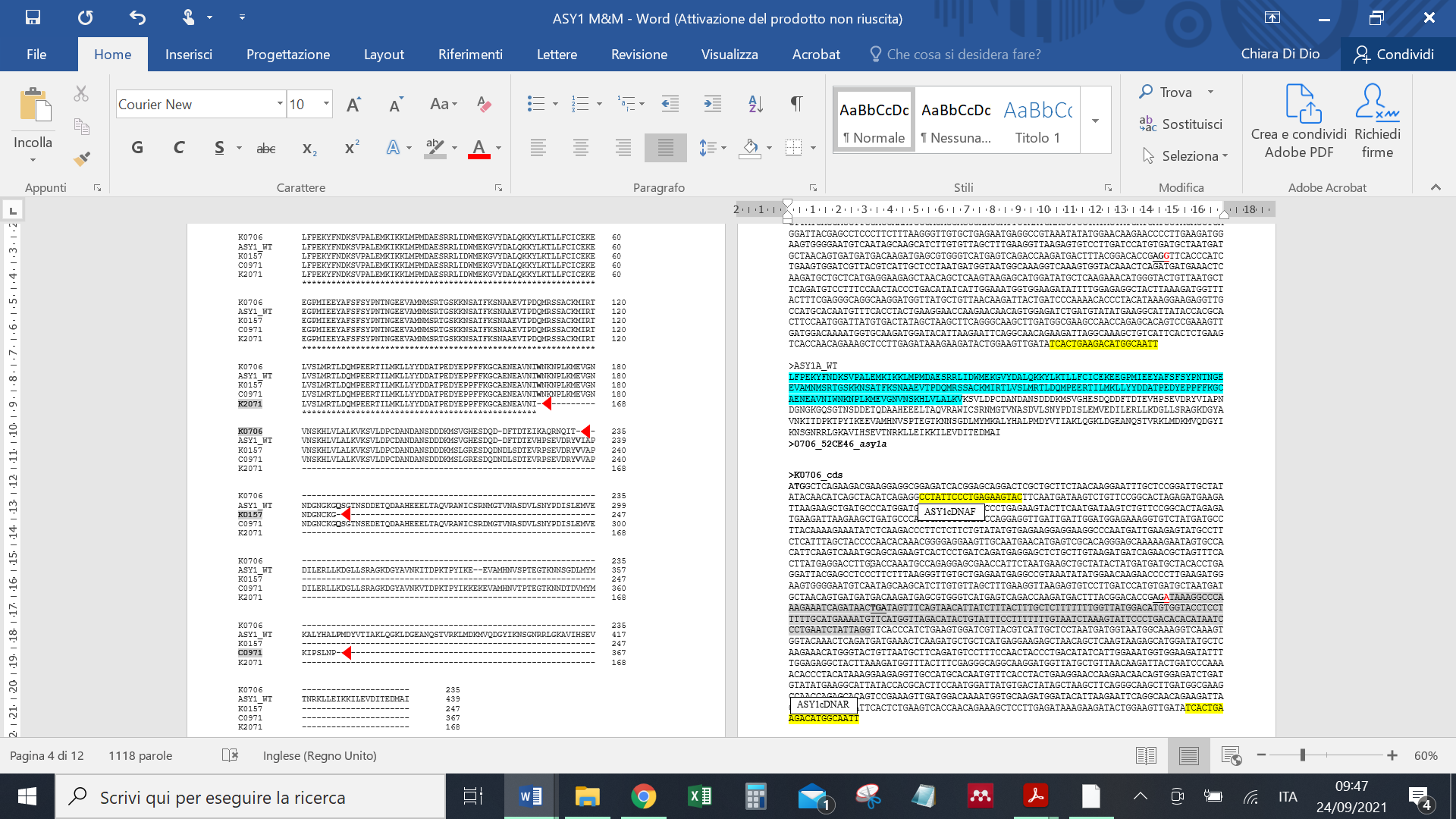


**Supplementary Figure 2*.* Clustal Ω multiple alignments of protein sequence from WT and predicted *asy1* TILLING mutant lines.** The alignment shows the corresponding position of the amino acid (in bold) affected by EMS treatment (red triangles). K0706= *asy1a*; K0157= *asy1b-1*; K2071= *asy1b-2*; C0971= *asy1 AAbbDD*; ASY1_WT= protein sequence of ASY1 wild-type.


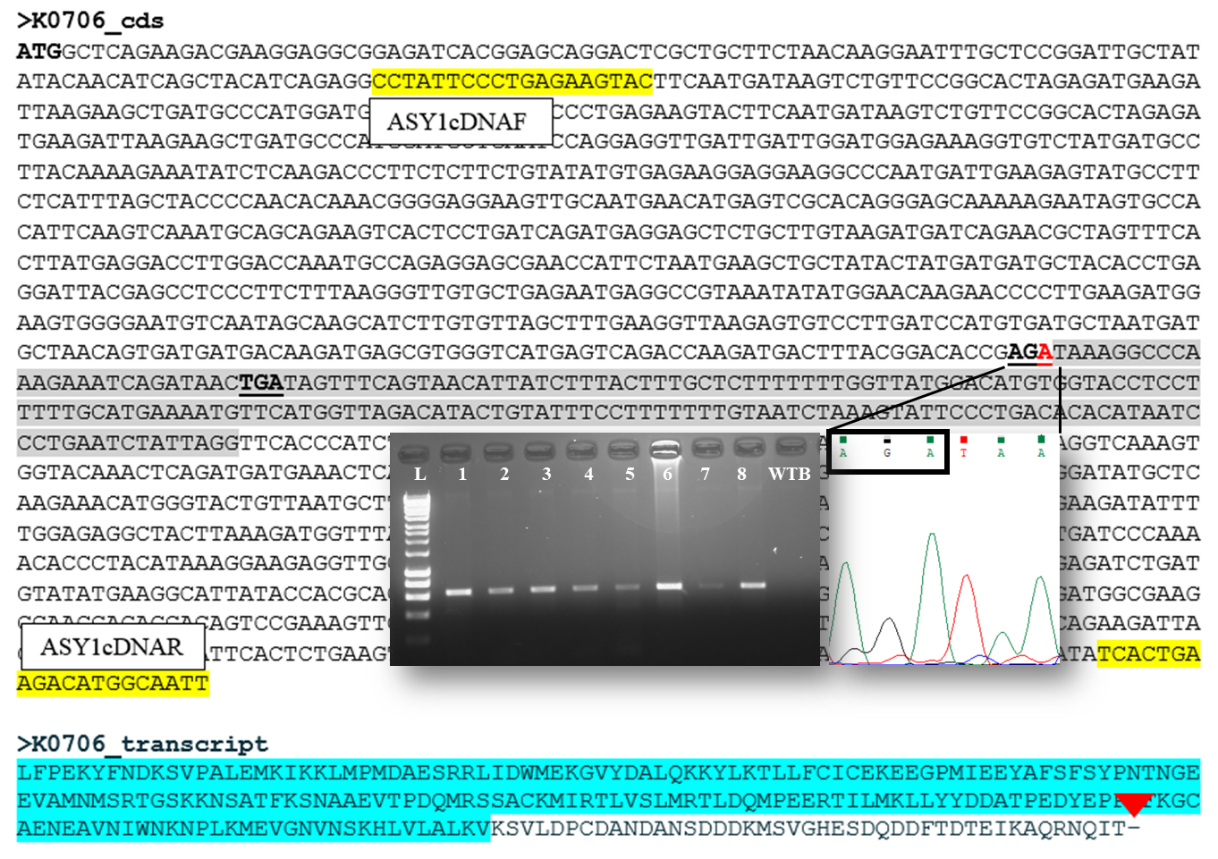


**Supplementary Figure 3. DNA coding region sequence of K0706 (*asy1a*) clone.** Splice donor site mutation AG|**G**T → AG|**A**T between exon 8 and exon 9, causing the retention of intron 8 at nucleotide position 714 (highlighted in grey) and predicted to generate a premature STOP codon TGA downstream is shown. Left insert represents the 600 bps PCR amplicons amplified by primer pairs specific for *TtASY1-5A* (L= DNA ladder; 1-8= *asy1a* cDNA clones; WTB = wild type Kronos B clone previously sequenced), while the insert on the right illustrates the sequence trace of the mutation site (image from Chromas Lite). Forward and reverse primers amplifying *ASY1* coding region sequence are labelled in yellow. The predicted translation product in reading frame 1 exemplifies the consequence of the TILLING mutation, a premature STOP codon (red triangle), in the respect of HORMA domain (highlighted in blue).


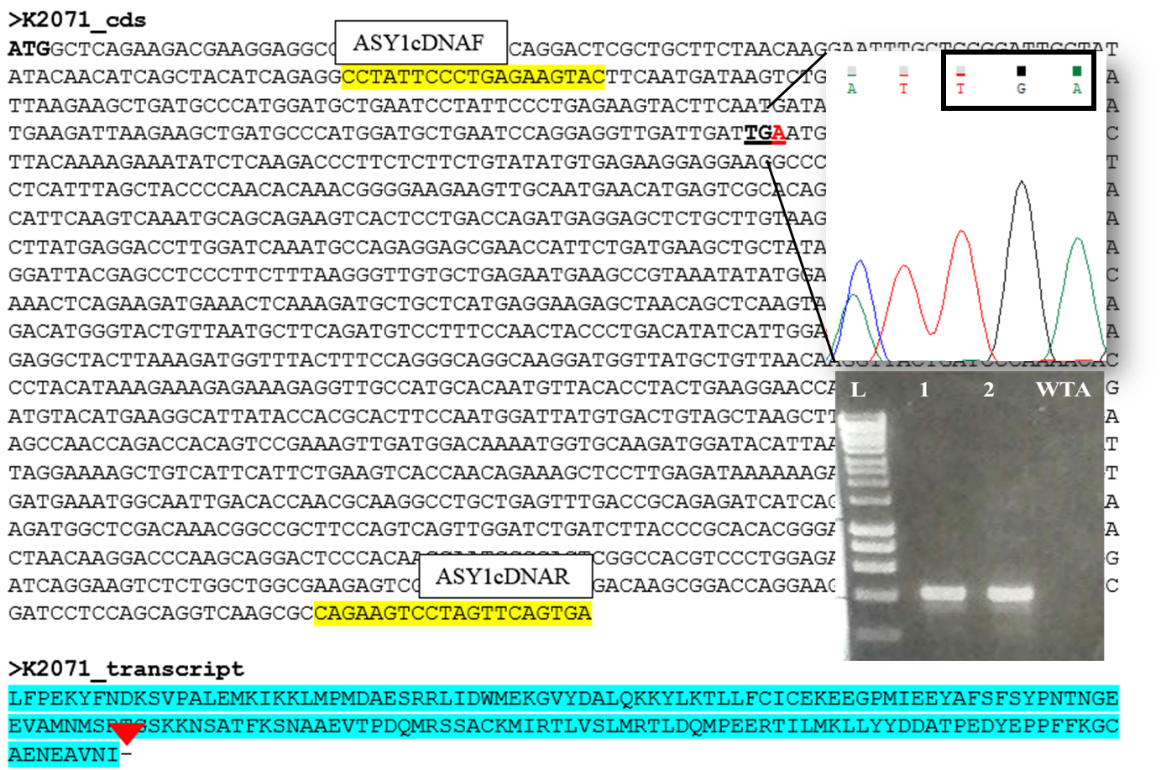


**Supplementary Figure 4. DNA coding region sequence of K2071 (*asy1b-2*) clone.** The translation TG**G** → TG**A** at nucleotide position 148, causing a premature STOP codon (TGA) is exemplified. Top insert shows the sequence trace of the STOP codon mutation (image from Chromas Lite), while the insert at the bottom illustrates the 400 bps PCR amplicons amplified by primer pairs specific for *TtASY1-5B* (L= DNA ladder; 1, 2= *asy1b-2* cDNA clones; WTA= wild type Kronos A clone previously sequenced). Forward and reverse primers amplifying *ASY1* coding region sequence are labelled in yellow. Translated protein (5'3' Frame 1 below) shows the STOP codon (red triangle) and the HORMA domain (highlighted in blue), missing 24 amino acid residues.


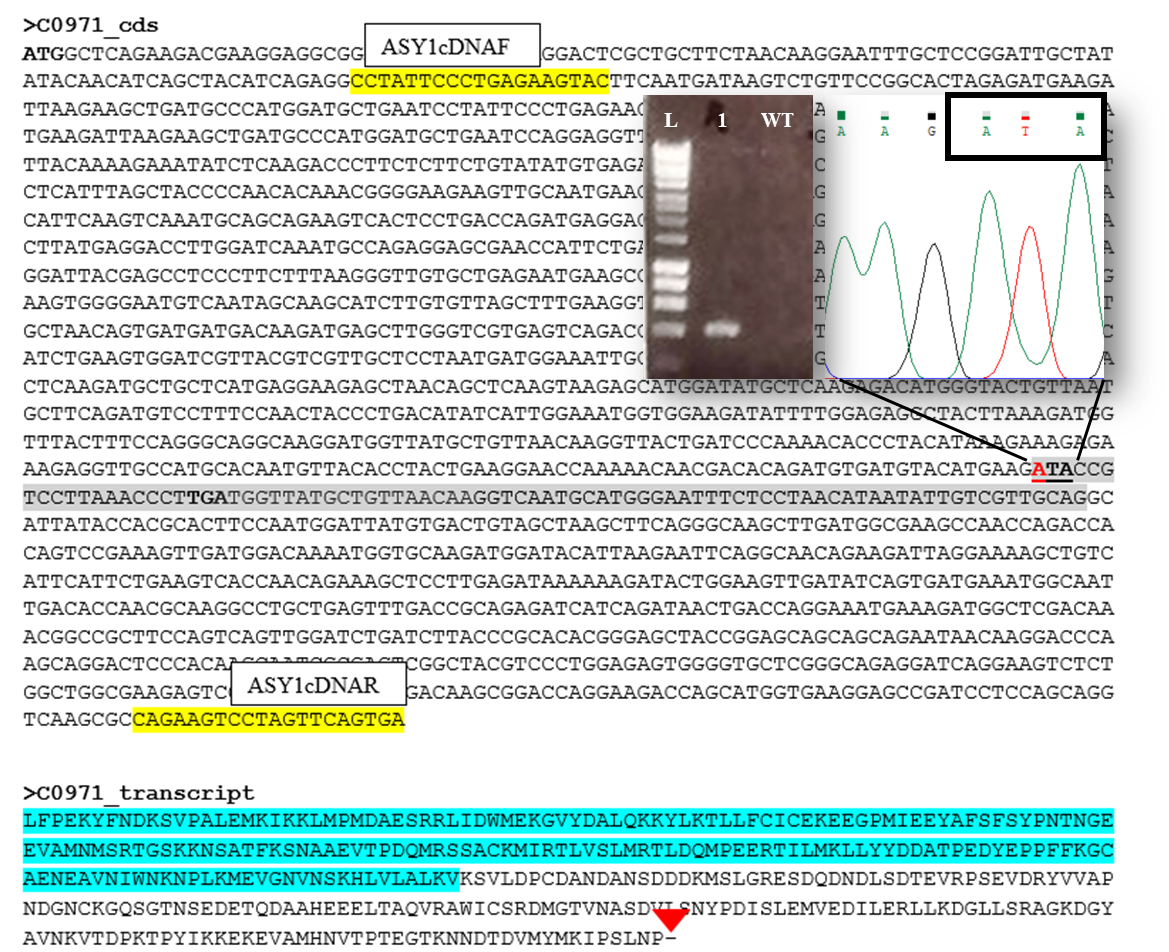


**Supplementary Figure 5.** **DNA coding region sequence of C0971 (*asy1 AAbbDD*) clone.** Splice donor site mutation **G**TA → **A**TA between exon 8 and exon 9, causing the retention of intron 9 at nucleotide position 1195 (highlighted in grey), predicted to generate a premature STOP codon TGA downstream, is represented. Left insert shows the 400 bps PCR amplicon amplified by primer pairs specific for *TaASY1-5B* (L= DNA ladder; 1= *asy1 AAbbDD* cDNA clone; WT= wild type Cadenza clone previously sequenced), while the insert on the right illustrates the sequence trace of the mutation site (image from Chromas Lite). Forward and reverse primers amplifying *ASY1* coding region sequence are labelled in yellow. Translated protein (5'3' Frame 1 below) shows the STOP codon (red triangle) and HORMA domain (highlighted in blue).


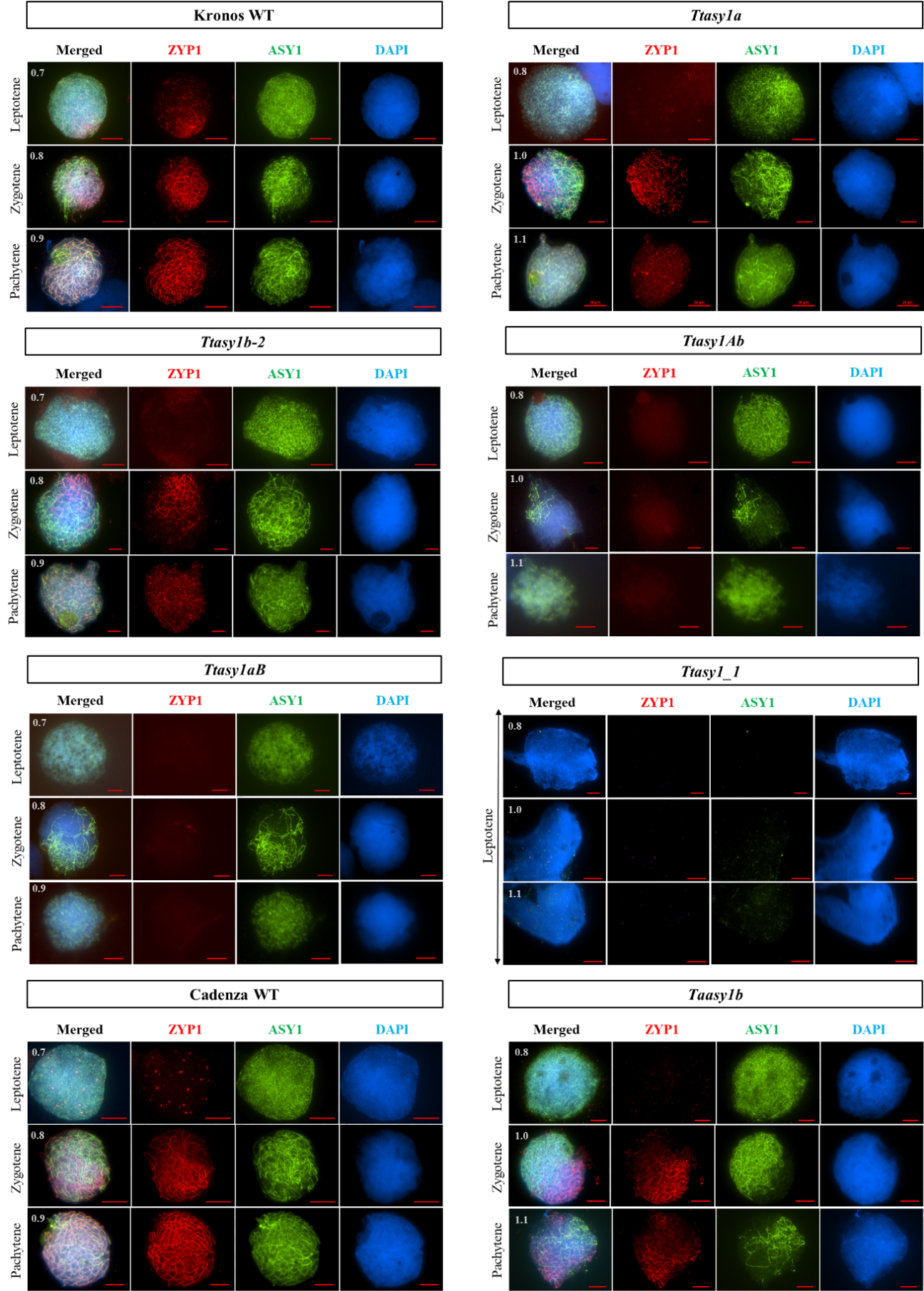


**Supplementary Figure 6. Immunolocalization of *Ta*ASY1 and *At*ZYP1 at meiotic prophase I stages in PMCs of Kronos WT, Cadenza WT and *asy1* mutants.** Merged images of transverse filament ZYP1 (red), chromosome axis ASY1 (green) and) chromatin counter-stained with DAPI (blue) are shown. In Kronos WT, axial elements were marked by continuous ASY1 labelling (green), and ZYP1 (red) began to linearize during leptotene; at zygotene, ZYP1 signal lengthened in regions of chromatin after ASY1 signal was unloaded, until fully linearized at synapsis points, thereby the SC connected the homologous chromosomes by pachytene. In *asy1* single KO in A and B sub-genomes, ASY1 (green) was still faithfully loaded onto the chromatin in a pattern similar to the WT until late leptotene, but ZYP1 (red) loading was largely delayed since the beginning of the leptotene; at zygotene, ZYP1 signals still appeared as punctuate foci in the nuclei, and small portion of SC showed ASY1 polycomplexes (intense green signals), marking clustering sites. At pachytene, ZYP1 (red) never fully synapsed, and ASY1 (green) was faint. In *Ttasy1* hypomorphic mutants (*Ab* and *aB*), ZYP1 was not detected in any meiotic stages, while ASY1 was unevenly distributed at leptotene and persisted during zygotene and pachytene, forming polycomplexes in asynapsed regions. The tripartite structure of SC appeared interrupted. Neither ASY1 or ZYP1 loaded on any *asy1 aabb* meiocytes, which are completely asyncronised. Analogously, in Cadenza WT, ASY1 (green) and ZYP1 (red) first appeared as faint punctate foci onto the same regions of the chromatin simultaneously during leptotene, albeit they never co-localise with one other; during zygotene, WT PMCs were marked by continuous ASY1 labelling, and ZYP1 formed short linear tracts, which lengthened and condensed; at pachytene, synapsis is complete with both ASY1 (green) and ZYP1 (red) overlapping. In *Taasy1b,* leptotene and zygotene showed WT-like ASY1 (green) formation, and a delay in ZYP1 (red) polymerization, which failed to properly elongate between the axial elements at pachytene, appearing with a diffuse and discontinuous signal. Anthers ranging from 0.7 to 1.1 mm in length, shown on the top left of merged images in Cadenza panels, correspond to distinct meiotic sub-stages. Scale bar = 10 µm (Kronos WT, *Ttasy1Ab, Ttasy1aB, Ttasy1_1, Taasy1b*) and 20 µm (*Ttasy1a, Ttasy1b-2*, Cadenza WT).

**Supplementary Table 1. Genotyping primers used to amplify ASY1 TILLING lines.**

| **Sub-genome Specific Primers** | **Oligonucleotide sequence 5’ → 3’** | **PCR conditions** |
| --- | --- | --- |
| ASY1gDNAaF | GCTGTTCTGATTCTGTCAC | [93_2:00_]^1^ [93_00:30_,56_00:45_,72_00:40_]^35^ [72_05:00_]^1^ |
| ASY1gDNA5aR | CATGGTTAGACATACTGTAT |  |
| K0706wt | GACTTTACGGACACCGAGG | [93_2:00_]^1^ [93_00:30_,66_00:45_,72_00:40_]^35^ [720_05:00_]^1^ |
| K0706common | CGCAGATGTGGTCTATATCC |  |
| K0706alt | GACTTTACGGACACCGAGA | [93_2:00_]^1^ [93_00:30_,64_00:45_,72_00:40_]^35^ [720_05:00_]^1^ |
| K0706common | CGCAGATGTGGTCTATATCC |  |
| ASY1gDNA5bF | GGCACTAGGTCAGGATCGG | [93_2:00_]^1^ [93_00:30_,54_00:45_,72_00:40_]^35^ [72_05:00_]^1^ |
| ASY1gDNA5bR | ACGTGTAGCGTATTAAGTTG |  |
| K2071wt | CCAGGAGGTTGATTGATTGG | [93_2:00_]^1^ [93_00:30_,64_00:45_,72_00:40_]^35^ [720_05:00_]^1^ |
| K2071common | ATGGCCTCATAAAGTGAAGG |  |
| K2071alt | CCAGGAGGTTGATTGATTGA | [93_2:00_]^1^ [93_00:30_,64_00:45_,72_00:40_]^35^ [720_05:00_]^1^ |
| K2071common | ATGGCCTCATAAAGTGAAGG |  |
| gASY1B_Cadenza_F | ATGCTGTTAACAAGGTCAAG | [93_2:00_]^1^ [93_00:30_,60_00:45_,72_00:40_]^35^ [72_05:00_]^1^ |
| gASY1B_Cadenza_R | GTAGCTAAGCTTCAGGG |  |
| C0971wt | GTACCGTCCTTAAACCCTTG | [93_2:00_]^1^ [93_00:30_,64_00:45_,72_00:40_]^35^ [720_05:00_]^1^ |
| C0971common | CAAGTTTTCTGGGCTGCTAT |  |
| C0971alt | ATACCGTCCTTAAACCCTTG | [93_2:00_]^1^ [93_00:30_,64_00:45_,72_00:40_]^35^ [720_05:00_]^1^ |
| C0971common | CAAGTTTTCTGGGCTGCTAT |  |

Primer pairs used for the amplification of *ASY1* genes were designed by aligning the reference sequences of *T. aestivum* ‘Chinese Spring’ using Clustal Omega. Sanger sequencing confirmed the predicted SNP mutation in the respective *ASY1* sub-genomes of the four TILLING lines selected and PCR-based genotyping allowed screening of the desired genotypes for this study. PCR primer sets flanking the corresponding SNP site were designed to individually amplify *ASY1-5A* and *ASY1-5B*. In tetraploid cv. Kronos, homozygous individuals aaBB (single KO in A sub-genome) and AAbb (single KO in B sub-genome) were found in K0706 (*asy1a*) and K2071 (a*sy1b-2*), respectively. Analogously, in hexaploid landrace Cadenza, homozygous individuals AAbbDD were identified in C0971 (*asy1 AAbbDD*).

**Supplementary Table 2. Comparing nucleotide and amino acid identities of polyploid wheat ASY1**

**Supplementary Table 3. ASY1 conserved structural domains predicted by Phyre2.**

**Supplementary Table 4. Seed counts from wild type and *asy1* TILLING lines.** A *t*-test two-sample distribution was applied to define the statistical significance (*P* < 0.05)

**Supplementary Table 5. Chi square test of meiotic prophase I stages and anther lengths comparing tetraploid and hexaploid *asy1* mutants with wild type.**

**Supplementary Table 6. Chiasma counts and meiotic metaphase I chromosome configurations in wild type Kronos**

**Supplementary Table 7. Chiasma counts and meiotic metaphase I chromosome configurations in *Ttasy1a***

**Table S8. Chiasma counts and meiotic metaphase I chromosome configurations in *Ttasy1b-1***

**Supplementary Table 9. Chiasma counts and meiotic metaphase I chromosome configurations in *Ttasy1b-2***

**Supplementary Table 10. Chiasma counts and meiotic metaphase I chromosome configurations in *Ttasy1Ab***

**Supplementary Table 11. Chiasma counts and meiotic metaphase I chromosome configurations in *Ttasy1aB***

**Supplementary Table 12. Chiasma counts and meiotic metaphase I chromosome configurations in *Ttasy1_1***

**Supplementary Table 13. Chiasma counts and meiotic metaphase I chromosome configurations in wild type and *Taasy1b* Cadenza**

**Supplementary Table 14. Chiasma counts of wild type Kronos (plant a)/*Ae. variabilis***

**Supplementary Table 15. Chiasma counts of wild type Kronos (anther b1)/*Ae. variabilis***

**Supplementary Table 16. Chiasma counts of wild type Kronos (anther b2)/*Ae. variabilis***

**Supplementary Table 17. Chiasma counts of *Ttasy1b-2* (anther a1)/*Ae. variabilis***

**Supplementary Table 18. Chiasma counts of *Ttasy1b-2* (anther a2)/*Ae. variabilis***

**Supplementary Table 19. Chiasma counts of *Ttasy1b-2* (anther a3)/*Ae. variabilis***
